# Comparable burden of severe acute respiratory illness among children and older adults in Kenya: evidence from a multi-site health utilization survey

**DOI:** 10.1016/j.ijregi.2026.101011

**Published:** 2026-09-13

**Authors:** Eric Osoro, Jeanette Dawa, Isaac Ngere, Nancy A. Otieno, Godfrey Bigogo, Mutono Nyamai, Samuel Waiguru, M. Kariuki Njenga, Bryan O. Nyawanda

**Affiliations:** 1 Washington State University Global Health Kenya, Nairobi, Kenya; 2 Paul G. Allen School for Global Health, Washington State University, Pullman, Washington, USA; 3 Center for Global Health Research, Kenya Medical Research Institute, Nairobi, Kenya; 4 Swiss Tropical and Public Health Institute, Allschwil, Switzerland; 5 University of Basel, Basel, Switzerland

**Keywords:** Health Utilization Surveys, Influenza, Severe Acute Respiratory Illness, Disability-Adjusted Life-Years, Kenya

## Abstract

**Objectives:**

Hospital-based influenza surveillance may underestimate the population-level respiratory disease burden in sub-Saharan Africa. Previous Kenyan estimates reported severe acute respiratory illness (SARI) rates among adults aged ≥65 years that were 4-10 times lower than those among children aged <5 years. We used community health utilization survey (HUS) data to estimate national and county-level HUS-defined SARI and influenza-associated SARI episode rates in Kenya, with adjustments for geographic variation and health care–seeking behavior.

**Methods:**

Data from 21,088 participants in a 2018 HUS conducted in Nakuru, Kakamega, and Siaya counties were included in the burden analysis. Age-specific HUS-defined SARI episode rates were extrapolated geographically using relative county lower respiratory infection disability-adjusted life-year factors from the Global Burden of Disease 2016 study. Age-specific proportions of episodes with actual admission or recommendation for hospitalization were obtained from the HUS, whereas the 2014 Kenya Demographic and Health Survey provided a relative county health care–seeking modifier. Influenza attribution used all-age positivity from seven surveillance sites or assigned regional sources during 2017-2019. Point estimates used observed or central inputs; 95% uncertainty intervals were derived from 10,000 design-aware resampling and joint uncertainty iterations.

**Results:**

The national HUS-defined SARI episode rate was 3318 (95% uncertainty interval: 2477-4326) per 100,000. Rates were 7758 (5008-11,156) among children aged <2 years, 7890 (5698-10,629) among children aged 2-4 years, and 7155 (3108-12,682) among adults aged ≥65 years, producing a U-shaped age distribution. The influenza-associated SARI rate was 365 (270-493) per 100,000.

**Conclusion:**

Among households with children aged <5 years, adults aged ≥65 years had a HUS-defined SARI burden comparable in magnitude to that of young children. The directly observed HUS age pattern persisted after national extrapolation. Influenza-associated age-specific estimates showed a similar pattern but were less directly supported by age-specific surveillance data. Community HUS data also identified substantial HUS-defined SARI that was not captured by sentinel hospital admissions.

## Highlights

• Survey-defined severe respiratory illness was high at both age extremes.
• Only 26% of actual admissions in the survey occurred at sentinel hospitals.
• The U-shaped age pattern persisted after national geographic extrapolation.
• Influenza-associated severe illness was estimated at 365 episodes per 100,000 annually.
• Influenza age patterns depended on all-age site or regional positivity estimates.

## Introduction

Severe acute respiratory illness (SARI) causes substantial global morbidity and mortality, with influenza virus contributing to 10%-12% of cases [1]. In the European Union and European Economic Area, influenza accounted for approximately 30% of the disability-adjusted life-year (DALY) burden among the 31 infectious diseases evaluated during 2009-2013 [2]. Although modeling studies suggest that low- and middle-income countries experience a higher influenza burden than high-income countries, country-level data remain limited due to incomplete surveillance infrastructure and underreporting [3].

Where robust burden estimates exist, they have enabled cost-effective interventions, including seasonal vaccination programs, with measurable reductions in morbidity and mortality [1,4]. Conversely, data gaps in sub-Saharan Africa have impeded recognition of influenza as a public health priority and delayed evidence-based intervention strategies [4]. Since the mid-2000s, sentinel surveillance for influenza-associated SARI has expanded across Africa, providing critical data on disease burden, circulating strains, and seasonal patterns [5]. However, these predominantly hospital-based systems can underestimate the population burden in settings where health-seeking behavior varies substantially across communities [[6], [7], [8]].

Health utilization surveys (HUSs) address this limitation by capturing disease episodes outside sentinel facilities. Previous Kenyan burden estimates were anchored to admissions captured at designated surveillance hospitals and extrapolated from one or a limited number of population-based surveillance sites, with additional assumptions about health care–seeking and geographic variation [[9], [10], [11]].

Previous burden estimates in Kenya and other sub-Saharan African countries may have underestimated the disease burden in adults aged ≥65 years. Studies from Kenya, Rwanda, and Zambia reported hospitalized and influenza-associated SARI rates among older adults that were 4- to 10-fold lower than the rates for children aged under 5 years [12,13]. This pattern contrasts sharply with findings from high-income countries, where older adults typically experience the highest burden of influenza-associated hospitalization [14,15]. The lower rates observed in African studies may partly reflect methodological differences, including reliance on single surveillance sites, assumptions about health care–seeking patterns, and incomplete capture of admissions outside designated surveillance facilities. Accurate age-specific burden estimates are essential to inform vaccination policy because they determine priority populations and enable modeling of the potential public health impact.

We used HUS data from catchment populations of three geographically diverse surveillance sites to estimate HUS-defined SARI episode rates, including episodes that resulted in actual hospital admissions, episodes for which hospitalization was recommended but did not occur, and episodes that met age-specific symptom or functional limitation severity criteria without admission or a hospitalization recommendation. We then extrapolated these estimates nationally and by county and estimated the influenza-associated component.

## Methods

### Study sites and population

For this burden analysis, we used data from the 2018 HUS collected among residents living within the catchment areas of sentinel influenza surveillance hospitals in Nakuru (urban), Kakamega (peri-urban), and Siaya (rural) counties in Kenya (Figure 1). Each county included an established sentinel influenza surveillance hospital supported through collaboration among the Ministry of Health, Kenya Medical Research Institute (KEMRI), and the US Centers for Disease Control and Prevention [16]. Catchment areas were defined by reviewing inpatient addresses from the 2-5 years preceding the survey and identifying the set of sublocations (the lowest administrative units) in which at least 80% of patients with SARI attending each surveillance hospital resided [17].

**Figure 1:**
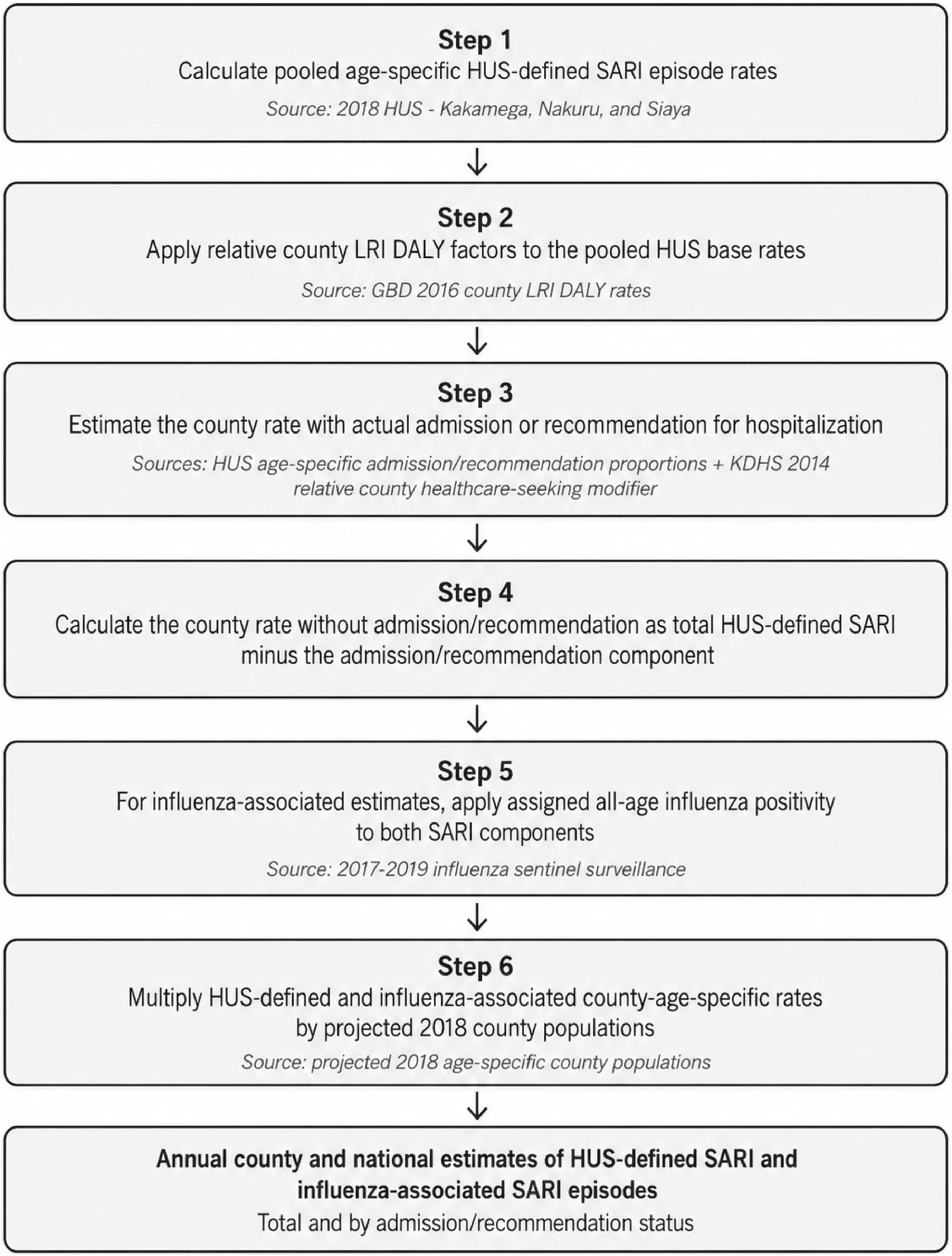
Map of Kenya showing influenza sentinel surveillance sites and the three HUS counties included in the burden analysis. Abbreviation: HUS, health utilization survey.

The HUS used a two-stage cluster sampling procedure, first selecting sublocations and then households. From these sublocations, a random subset was selected, and the required households were allocated proportionately to population size using the 2009 census. Random spatial coordinates were generated within the selected sublocations; survey teams located each coordinate and screened the nearest household. Only households with a child aged <5 years were eligible; if no eligible household was found, the team proceeded to the next coordinate. Human immunodeficiency virus prevalence in 2018 was 3.0% in Nakuru, 3.9% in Kakamega, and 15.3% in Siaya (national average: 4.9%) [18].

A structured questionnaire collected household- and individual-level information on pneumonia episodes in the preceding 12 months and acute febrile, diarrheal, or respiratory illnesses in the preceding 14 days, including symptoms, health care services accessed, hospitalization status, and health care provider recommendations.

### Sample size estimation

Based on previous western Kenya surveillance data showing a 2.1% pneumonia prevalence and a 16% hospitalization proportion for pneumonia [19], the sample size calculation assumed an average of four persons per household and 10% precision, yielding a requirement of 620 households to identify at least 52 persons with self-reported pneumonia. With a design effect of 2 and a 15% allowance for nonresponse, the target was 1450 households per county.

### Study definitions

Pneumonia was defined as (i) cough for >2 days and difficult breathing for >2 days or (ii) a diagnosis of pneumonia by a health care professional (doctor, clinical officer, or nurse) in the 12 months preceding the survey [20].

HUS-defined SARI was pneumonia that met at least one severity pathway: actual hospital admission, a health care professional recommendation for hospitalization, or age-specific severity criteria in participants who were not admitted and had not received a recommendation for admission. For burden modeling, episodes with either actual admission or a recommendation for hospitalization were grouped as “SARI with admission or recommendation for hospitalization.” This category includes actual admissions and recommendation-only episodes. Actual admission was defined as at least one overnight inpatient stay. Remaining HUS-defined SARI episodes were classified as “SARI without admission or recommendation for hospitalization.”

For episodes without admission or a hospitalization recommendation, severity was determined using age-specific criteria: for children aged <5 years, any one of the inability to drink or eat, vomiting everything, convulsions, or loss of consciousness; for persons aged ≥5 years, “limited a lot” responses to at least two functional limitation questions listed in Supplementary Table 1 [21,22]. These symptom- and function-based criteria form an operational community severity construct and have not been clinically validated against the World Health Organization (WHO) hospitalized SARI case definition.

#### Calculation of health utilization survey–defined severe acute respiratory illness and influenza-associated severe acute respiratory illness episode rates

We applied a six-step framework to estimate national and county-level HUS-defined SARI and influenza-associated SARI episode rates (Figure 2; detailed in Supplementary Methods). Pooled age-specific base episode rates were calculated from HUS participants in Nakuru, Kakamega, and Siaya, with recurrent episodes during the 12-month recall period retained in the numerator. For geographic extrapolation, county-specific lower respiratory infection (LRI) DALY rates from Global Burden of Disease (GBD) 2016 [23] were used to construct relative geographic scaling factors (Supplementary Table 2) (Achoki et al. [23], 2019; Supplementary Table 2). The approach assumes that the relative county variation in HUS-defined SARI burden is reasonably approximated by the relative county variation in modeled LRI DALY burden.

**Figure 2:**
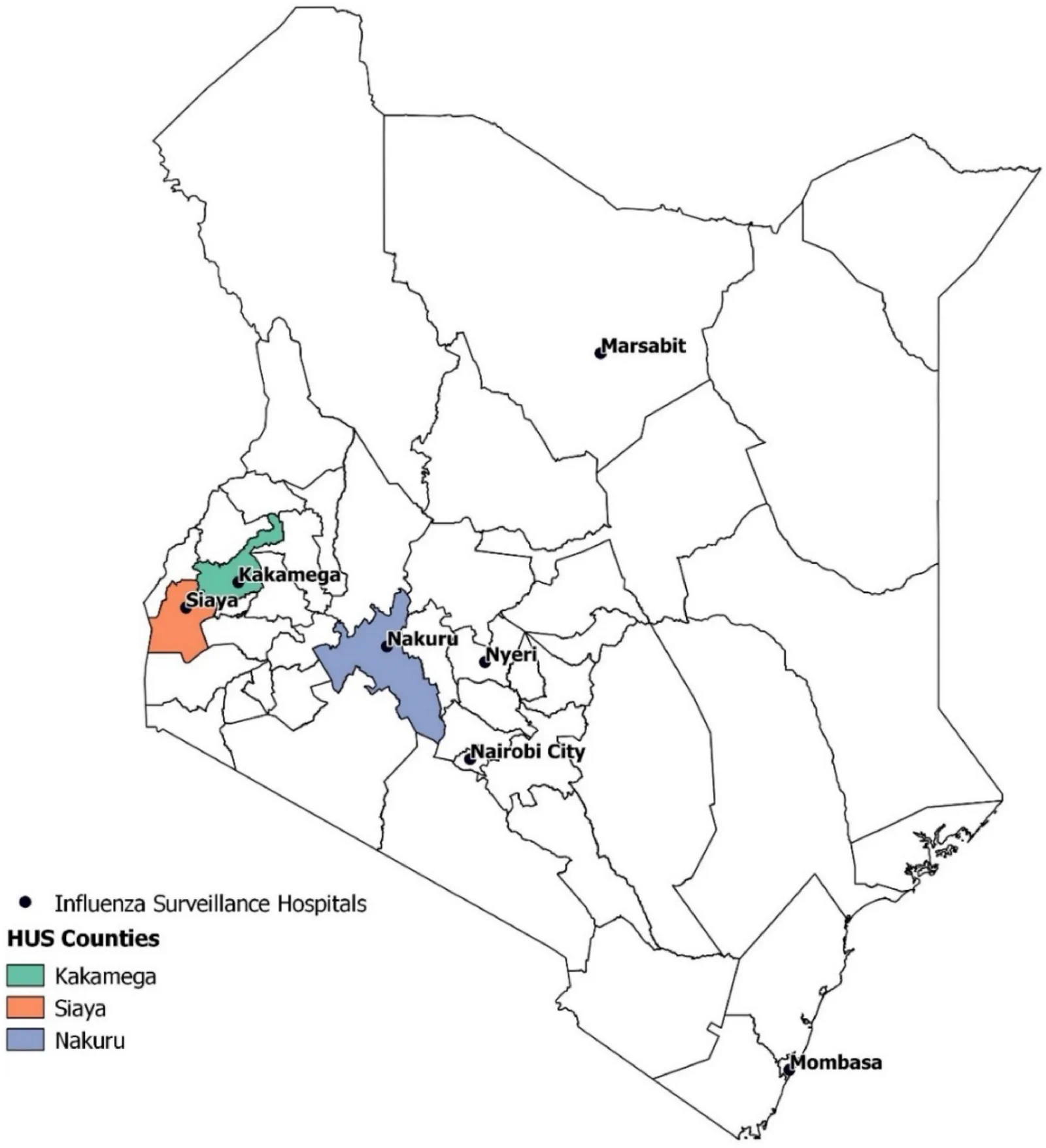
Analytic framework for estimating county and national HUS-defined SARI and influenza-associated SARI burden in Kenya. Pooled age-specific HUS-defined SARI rates from the 2018 HUS in Kakamega, Nakuru, and Siaya were scaled to counties using relative GBD 2016 lower respiratory infection (LRI) disability-adjusted life-year (DALY) rates. Rates were partitioned into admission/recommendation and no-admission/recommendation components using HUS age-specific proportions and a relative county health care–seeking modifier from KDHS 2014. For influenza-associated estimates, assigned 2017-2019 all-age influenza positivity was applied to both components. County age-specific rates were then multiplied by projected 2018 populations to obtain annual county and national episode estimates. Admission/recommendation denotes actual hospital admission or clinician recommendation for hospitalization. Abbreviations: GBD, Global Burden of Disease; HUS, health utilization survey; KDHS, Kenya Demographic and Health Survey; SARI, severe acute respiratory illness.

The HUS provided age-specific proportions of SARI episodes that resulted in actual admission or a recommendation for hospitalization. For geographic extrapolation, 2014 Kenya Demographic and Health Survey (KDHS) data on health care–seeking for acute respiratory illness among children aged <5 years [24] were used to construct a relative county health care–seeking modifier: each county’s weighted care-seeking probability was divided by the pooled probability in Kakamega, Nakuru, and Siaya. Because comparable county-level estimates were unavailable for older ages, this relative geographic pattern was applied across age groups, whereas age-specific admission or recommendation proportions remained HUS-derived. SARI without admission/recommendation was calculated as total HUS-defined SARI minus the admission or recommendation component.

Influenza attribution used surveillance data from seven observed sites—Siaya, Kakamega, Mombasa, Nakuru, Nyeri, Kenyatta National Hospital, and Marsabit—during 2017-2019 (Figure 1). Age-specific positivity was examined, but surveillance sampling among persons aged ≥5 years was sparse, particularly within individual sites and older age groups; all-age positivity from the assigned surveillance site or regional composite was, therefore, applied across age groups. The same assigned positivity was applied to the admission or recommendation and no-admission/recommendation components. County-specific episode numbers were calculated using projected 2018 county populations derived from the 2009 census and annual population growth assumptions [25]. A summary of the data sources and thier application in the burden model is summarized in Supplementary Table 3.

#### Uncertainty intervals

Point estimates were calculated from observed HUS data and central model inputs. Uncertainty was propagated with 10,000 joint iterations. HUS uncertainty used a two-stage cluster bootstrap that resampled sublocations and then households; KDHS modifiers used a stratified primary sampling unit bootstrap respecting the survey design; GBD geographic factors were sampled coherently with a shared pooled denominator for Kakamega, Nakuru, and Siaya; and influenza uncertainty used bounded site/regional positivity draws shared across counties assigned to the same source. The 2.5th and 97.5th percentiles of the resulting distributions are reported as 95% uncertainty intervals (UIs). Analyses were conducted in R.

### Ethical considerations

The study received approval from the Kenya Medical Research Institute (KEMRI) Scientific & Ethics Review Unit (SERU #3667), Washington State University (#16655), and US Centers for Disease Control and Prevention (7130) Institutional Review Boards. Written informed consent was obtained from household heads/main respondents and from participating adult household members; assent was obtained from minors aged ≥13 years.

### Patient and public involvement

Members of the public participated as respondents in the HUS, which generated primary data for this analysis. They were not involved in the design, analysis, interpretation, or dissemination of this research.

## Results

### Health utilization survey enrollment and health utilization survey–defined severe acute respiratory illness episodes

The burden analysis included 21,088 HUS participants from 4054 households across Siaya (n = 7451), Kakamega (n = 7383), and Nakuru (n = 6254) counties. The largest proportion of participants (43.6%) belonged to the 15-49 years age group, whereas those aged <2 years and ≥65 years constituted 7.6% and 1.7% of the participants, respectively.

Among 21,088 analytic participants, 729 reported at least one HUS-defined SARI episode during the 12-month recall period. Because recurrent episodes were retained for burden estimation, 889 episodes contributed to the annual episode rate. Of these, 262 episodes involved either actual hospital admission or a health care professional recommendation for hospitalization: 200 were actual admissions and 62 were recommendation-only episodes. Among the 200 actual admissions, 52 (26.0%) occurred at designated sentinel surveillance hospitals and 148 (74.0%) occurred at other hospitals. Recommendation-only episodes accounted for 23 of 137 (16.8%) of the admission or recommendation category among children aged <5 years, 24 of 62 (38.7%) among adults aged 15-49 years, and three of 10 (30.0%) among adults aged ≥65 years.

### Base health utilization survey–defined severe acute respiratory illness episode rates

Annual HUS-defined SARI episode rates per 100,000 participants were 3100 (95% UI: 2307-4060) in Siaya, 4077 (3050-5349) in Kakamega, and 5708 (4217-7427) in Nakuru, with a pooled rate of 4216 (3528-4980) (Supplementary Table 4). The pooled age-specific rates were 9857 among children aged <2 years, 10,025 among children aged 2-4 years, 2063 among those aged 5-14 years, 2351 among adults aged 15-49 years, 4406 among adults aged 50-64 years, and 9091 among adults aged ≥65 years, demonstrating a U-shaped age pattern before national extrapolation. Overall, 262 of 889 (29.5%) HUS-defined SARI episodes resulted in actual admission or a recommendation for hospitalization.

#### National and county annual health utilization survey–defined severe acute respiratory illness episode rates

The national annual HUS-defined SARI episode rate was 3318 (95% UI: 2477-4326) per 100,000, with county point estimates ranging from 2069 in Laikipia to 5683 in Nakuru (Supplementary Table 5A; Supplementary Figure 1).

The U-shaped age distribution was retained after national extrapolation. Annual HUS-defined SARI episode rates per 100,000 were 7758 (95% UI: 5008-11,156) among children aged <2 years, 7890 (5698-10,629) among children aged 2-4 years, 1624 (1040-2354) among those aged 5-14 years, 1851 (1302-2493) among adults aged 15-49 years, 3467 (1746-5651) among adults aged 50-64 years, and 7155 (3108-12,682) among adults aged ≥65 years (Supplementary Table 5A).

SARI with admission or recommendation for hospitalization had a national rate of 950 (95% UI: 653-1323) per 100,000. SARI without admission/recommendation accounted for a rate of 2368 (1719-3169) per 100,000, approximately 71.4% of the national point estimate. Across counties, the proportion classified as SARI without admission/recommendation ranged from 59.3% to 85.4% (Supplementary Tables 5B and C).

#### Influenza-associated burden of severe acute respiratory illness

Across the 2017-2019 surveillance inputs, the influenza attribution data comprised 1521 tested and 172 influenza-positive counts across seven observed surveillance sites, corresponding to an 11.3% positivity rate (Supplementary Table 6). The national influenza-associated SARI episode rate was 365 (95% UI: 270-493) per 100,000, with county point estimates ranging from 212 in Marsabit to 796 in Nakuru (Table 1; Supplementary Table 7A). The national admission or recommendation component was 104 (72-149) per 100,000, and the no-admission/recommendation influenza-associated SARI component was 261 (187-359) per 100,000.

**Table 1:** National and county annual rates and estimated numbers of influenza-associated HUS-defined SARI episodes, Kenya, 2018.

National/County	Influenza-associated SARI with actual admission or recommendation for hospitalization rate per 100,000 (95% UI)	No admission/recommendation influenza-associated SARI rate per 100,000 (95% UI)	Total influenza-associated SARI rate per 100,000 (95% UI)	Estimated annual number of influenza-associated SARI episodes (95% UI)
KENYA	104 (72-149)	261 (187-359)	365 (270-493)	178,292 (131,878-241,000)
Baringo	103 (57-171)	252 (151-390)	356 (220-542)	2499 (1545-3810)
Bomet	91 (46-156)	238 (139-374)	330 (200-505)	3022 (1835-4624)
Bungoma	76 (37-134)	277 (153-455)	353 (197-576)	6134 (3427-10,024)
Busia	193 (102-330)	337 (184-557)	530 (300-856)	4986 (2822-8053)
Elgeyo-Marakwet	147 (87-234)	214 (128-331)	361 (229-539)	1688 (1071-2524)
Embu	159 (88-273)	263 (151-425)	422 (256-666)	2757 (1675-4352)
Garissa	37 (0-107)	209 (71-459)	246 (85-523)	1937 (668-4124)
Homabay	151 (86-255)	359 (216-589)	510 (314-828)	6212 (3822-10,090)
Isiolo	74 (24-165)	195 (67-406)	270 (94-553)	489 (170-1003)
Kajiado	105 (59-169)	282 (176-428)	387 (247-575)	3364 (2148-4997)
Kakamega	131 (67-228)	357 (196-588)	488 (276-782)	10,256 (5806-16,428)
Kericho	99 (54-169)	249 (147-387)	348 (213-529)	3339 (2039-5075)
Kiambu	134 (79-221)	273 (173-427)	407 (272-614)	8359 (5591-12,609)
Kilifi	82 (44-142)	248 (152-397)	330 (205-522)	4630 (2883-7330)
Kirinyaga	69 (0-208)	396 (230-626)	464 (305-715)	3100 (2040-4775)
Kisii	69 (35-127)	178 (101-301)	247 (144-409)	3595 (2097-5960)
Kisumu	129 (67-237)	257 (141-444)	387 (220-650)	4737 (2702-7964)
Kitui	70 (36-124)	210 (122-336)	280 (165-442)	3590 (2118-5666)
Kwale	84 (44-153)	237 (143-387)	322 (199-518)	2646 (1637-4255)
Laikipia	92 (49-157)	198 (117-314)	290 (179-446)	1463 (903-2251)
Lamu	136 (75-237)	321 (196-517)	457 (288-722)	587 (371-927)
Machakos	76 (40-133)	246 (150-385)	323 (201-497)	4486 (2799-6901)
Makueni	92 (48-159)	236 (135-381)	327 (194-523)	3662 (2168-5854)
Mandera	70 (0-181)	207 (67-467)	278 (93-604)	3604 (1205-7834)
Marsabit	60 (17-133)	153 (51-328)	212 (73-441)	782 (268-1624)
Meru	205 (118-332)	304 (179-475)	509 (318-770)	8730 (5458-13,206)
Migori	155 (86-266)	323 (189-533)	479 (293-771)	5552 (3401-8949)
Mombasa	122 (65-213)	245 (146-403)	367 (229-582)	4357 (2719-6910)
Muranga	153 (91-251)	299 (193-467)	453 (308-684)	5398 (3675-8151)
Nairobi	70 (39-118)	177 (110-275)	247 (159-376)	9787 (6319-14,906)
Nakuru	248 (127-397)	548 (324-854)	796 (496-1178)	16,257 (10,138-24,074)
Nandi	93 (52-155)	233 (142-360)	326 (206-493)	3105 (1964-4693)
Narok	113 (64-185)	268 (164-408)	381 (242-573)	4102 (2601-6162)
Nyamira	80 (35-170)	207 (113-347)	287 (173-466)	2173 (1307-3529)
Nyandarua	142 (58-279)	483 (305-751)	626 (422-940)	4720 (3181-7090)
Nyeri	180 (103-294)	387 (254-595)	567 (390-846)	4976 (3418-7418)
Samburu	96 (44-177)	304 (178-481)	400 (242-620)	1133 (684-1755)
Siaya	93 (45-173)	190 (105-328)	283 (162-475)	3012 (1730-5061)
Taita taveta	143 (82-243)	269 (165-431)	413 (263-645)	1487 (948-2323)
Tana River	143 (80-252)	324 (193-534)	468 (288-755)	1420 (874-2292)
Tharaka-nithi	123 (67-207)	298 (179-467)	421 (261-649)	1945 (1208-2999)
Trans Nzoia	113 (62-183)	219 (130-343)	332 (204-504)	3437 (2115-5217)
Turkana	66 (20-152)	173 (59-366)	239 (82-496)	2586 (889-5366)
Uasin Gishu	88 (45-146)	291 (178-439)	378 (238-562)	4280 (2688-6356)
Vihiga	148 (73-275)	398 (211-676)	546 (296-925)	3834 (2075-6491)
Wajir	36 (9-99)	211 (70-472)	247 (84-549)	2065 (702-4593)
West Pokot	101 (55-172)	199 (114-317)	300 (179-469)	1948 (1160-3045)

Admission or recommendation includes episodes with either actual hospital admission or a health care professional’s recommendation for hospitalization. No admission/recommendation denotes episodes with neither actual admission nor a recommendation for hospitalization.

Abbreviations: HUS, health utilization survey; SARI, severe acute respiratory illness; UI, uncertainty interval.

Age-specific modeled influenza-associated SARI rates were highest at the two age extremes, but these estimates additionally depend on the assumption that the assigned all-age surveillance site or regional positivity applies across age groups (Figure 3; Supplementary Table 7A). Nationally, an estimated 178,292 (95% UI: 131,878-241,000) influenza-associated SARI episodes occur annually; the largest county totals were estimated in Nakuru (16,257) and Kakamega (10,256). (Figure 4)

**Figure 3:**
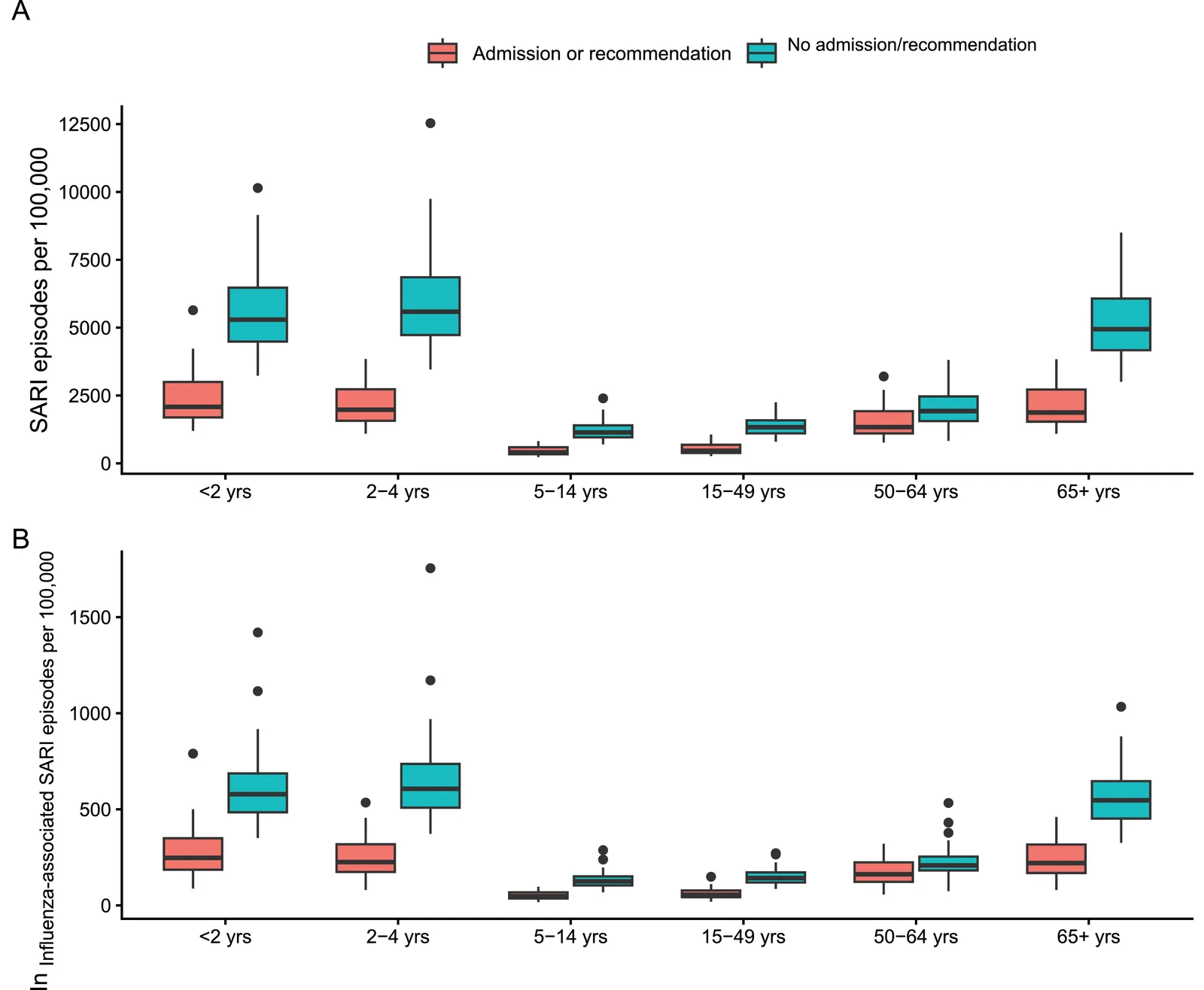
Age-specific HUS-defined SARI and influenza-associated SARI episode rates by admission/recommendation status across counties, Kenya. (A) HUS-defined SARI. (B) Influenza-associated SARI. Box plots show the distribution of county-level point estimates. Admission/recommendation denotes SARI episodes with either actual hospital admission or a clinician recommendation for hospitalization. No admission/recommendation denotes SARI episodes with neither actual hospital admission nor a clinician recommendation for hospitalization. Abbreviations: HUS, health utilization survey; SARI, severe acute respiratory illness.

**Figure 4:**
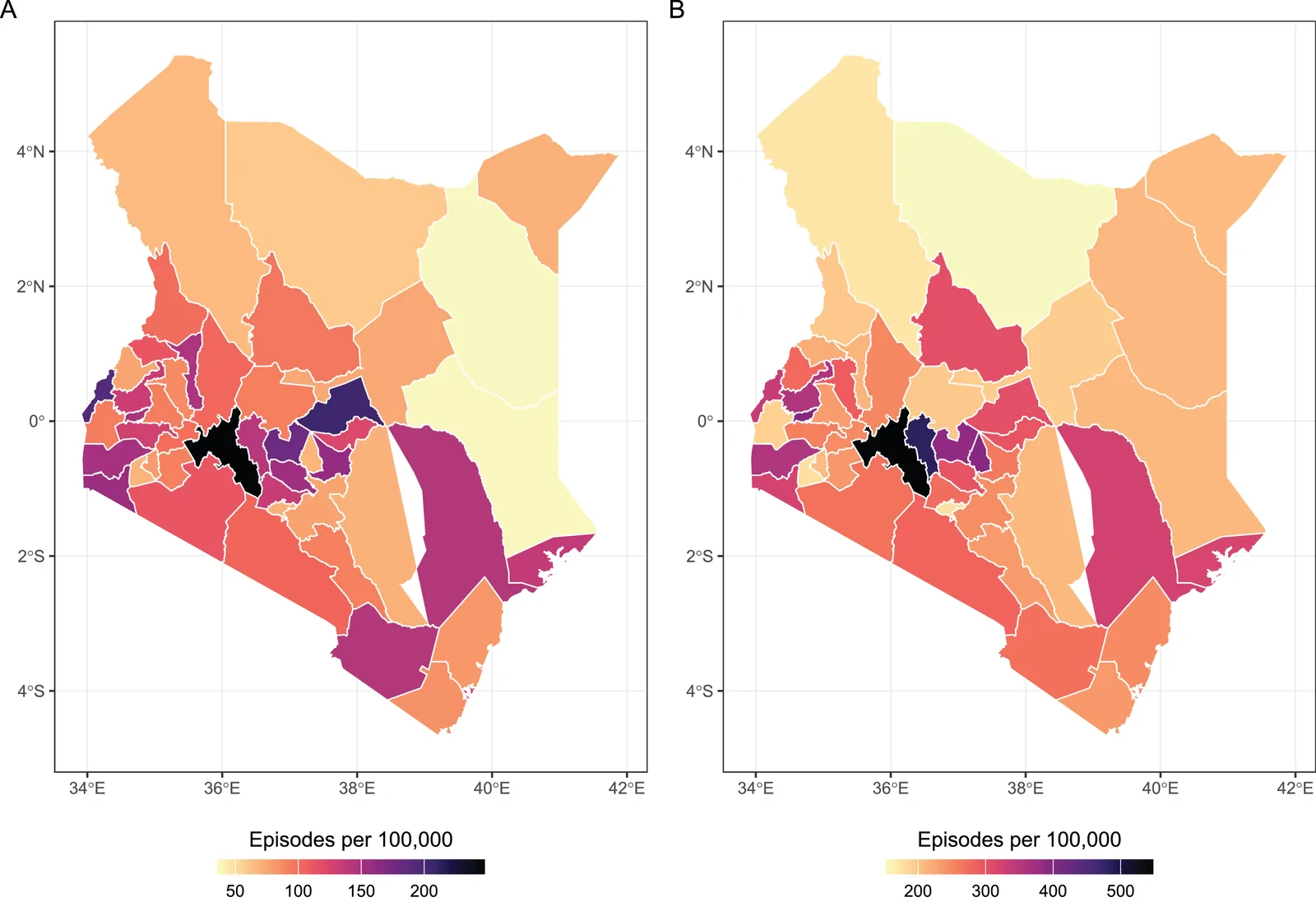
Influenza-associated severe acute respiratory illness (SARI) episode rates by county, Kenya, 2018. (A) Influenza-associated SARI with actual admission or recommendation for hospitalization. (B) Influenza-associated SARI without admission or recommendation for hospitalization. Choropleth maps show county-level point estimates per 100,000 population.

## Discussion

Children aged <5 years and adults aged ≥65 years had a comparable modeled influenza-associated SARI burden, contrasting with previous Kenyan estimates [10]. The U-shaped distribution of HUS-defined SARI episode rates was directly observed in the HUS before geographic or influenza adjustments were made. The national extrapolation retained this pattern, whereas age-specific influenza-associated estimates additionally depend on applying site/regional influenza positivity across age groups. This age pattern resembles that reported in high-income settings [14,15] but differs from much of the published sub-Saharan African surveillance literature [12,13].

The comparable burden between young children and older adults differs from previous Kenyan estimates. The 2012-2014 national assessment reported rates among adults ≥65 years that were 4- to 10-fold lower than the rates for children <5 years [10], derived from single-site–based estimates extrapolated nationally. In the pooled HUS data from urban, peri-urban, and rural settings, young children and adults aged ≥65 years had similarly high HUS-defined SARI episode rates.

Direct comparison with previous sub-Saharan African burden estimates is complicated by differences in case definitions, surveillance design, health care–seeking adjustments, and whether estimates are anchored to hospital admissions or community-reported severe illness. One reason is that earlier surveillance-based approaches, including the approach described by Fuller et al. [11], anchor hospitalized incidence to admissions captured at designated surveillance hospitals and then estimate disease occurring outside hospital using health care utilization adjustments, whereas the present HUS directly identifies actual admissions at sentinel and non-sentinel hospitals, recommendation-only episodes, and other community episodes meeting the HUS severity criteria. The estimates presented here should, therefore, be interpreted in relation to the HUS-defined SARI construct and the modeling framework used in this analysis.

Previous national studies in Rwanda and Zambia reported substantially higher rates among young children than among older adults [12,13]. Our HUS findings also identify mechanisms by which hospital-based surveillance can under-ascertain severe illness. Although the HUS sampling frame was constructed from sublocations in which at least 80% of patients with SARI attending the respective sentinel hospitals resided, only 52 of 200 (26.0%) actual admissions reported in the HUS occurred at those sentinel hospitals; 148 (74.0%) occurred at other hospitals. In addition, 62 HUS-defined SARI episodes involved a recommendation for hospitalization but no actual admission. Thus, a sentinel admission approach can miss severe episodes that never culminate in admission and admissions occurring outside the sentinel network.

Approximately 71% of the modeled influenza-associated SARI burden was classified as SARI without admission/recommendation, although this proportion was not uniform across age groups. In the HUS, recommendation-only episodes accounted for 23 of 137 (16.8%) of the admission/recommendation category among children aged <5 years, 24 of 62 (38.7%) among adults aged 15-49 years, and three of 10 (30.0%) among adults aged ≥65 years. Failure to complete a recommended admission may reflect financial, access, or other barriers to hospital care [7,8,19].

Individuals with severe respiratory illness who do not reach a hospital may not receive a pathogen-specific diagnosis or hospital-level supportive care. Verbal autopsy studies in coastal Kenya have identified acute respiratory infections among the important causes of home death [26].

Sentinel hospital surveillance remains essential for virologic monitoring, vaccine effectiveness assessment, and detection of circulating strains, but population burden estimation may require periodic community HUS. Our framework uses community HUS data to estimate HUS-defined SARI episode rates as the basis for geographic and health care–seeking adjustments and influenza attribution. The framework encompasses severe episodes outside of sentinel hospital admissions, including recommendation-only episodes and admissions at non-sentinel hospitals.

County-level estimates varied substantially across Kenya. The geographic pattern reflects the combined effects of the LRI DALY scaling factors, county health care–seeking modifiers, and assigned influenza positivity inputs that were used in the burden model.

The comparable HUS-defined SARI burden at both age extremes supports the consideration of both groups in vaccination policy analyses. The current WHO guidelines include young children and older adults among priority groups for seasonal influenza vaccination [27]. Our national and county estimates provide locally derived burden inputs for such policy and cost-effectiveness analyses [9]. Age-specific influenza-associated estimates are more assumption-dependent than the underlying SARI estimates because site/regional all-age positivity was applied across age groups.

Several methodological limitations warrant consideration. First, HUS-defined SARI depended on 12-month recall of pneumonia episodes, hospitalization, and clinician recommendations, creating potential for recall error and telescoping. For severe episodes without admission or recommendation, age-specific danger sign or functional limitation criteria were used; among persons aged ≥5 years, these included self-reported functional limitations adapted from established instruments [21,22]. These criteria have not been clinically validated as equivalent to WHO hospitalized SARI, and subjective interpretation, severity misclassification, or misclassification of chronic respiratory conditions as acute pneumonia could affect episode rates.

Second, the sentinel surveillance case definition used for influenza testing (fever or history of fever, cough, recent onset, and hospitalization) [28] is more specific than the HUS community pneumonia/severity construct. Applying surveillance positivity to HUS-defined SARI, therefore, assumes etiologic comparability and could overestimate influenza attribution if positivity is systematically higher in hospitalized patients with fever who are under surveillance. Age-specific positivity was examined but not used because surveillance sampling among older persons was sparse: across the seven sites, only 11 specimens were tested among persons aged 50-64 years and nine among those aged ≥65 years. Even after combining all persons aged ≥5 years, site-specific denominators remained small, producing unstable age-specific site estimates. We, therefore, applied the all-age positivity from each assigned surveillance site or regional source across age groups; age-specific influenza-associated estimates should consequently be interpreted with greater caution than the underlying HUS-defined SARI estimates. Third, KDHS under-five acute respiratory illness care-seeking data supplied the relative county geographic modifier, while age-specific admission/recommendation proportions remained HUS-derived. Because comparable county-level estimates were unavailable for older ages, the relative geographic pattern observed among children aged <5 years was transferred across age groups. Health care–seeking behavior varies with age and context [7,8,19], so this geographic transferability assumption may not hold uniformly. Fourth, the GBD adjustment assumes that relative county variation in modeled LRI DALY burden approximates relative geographic variation in HUS-defined SARI; this proxy has not been independently validated. Fifth, household eligibility required a child aged <5 years, so adults aged ≥65 years were sampled only from such households; the older-adult sample was small (n = 363), contributing to wider uncertainty and limiting generalizability to all older adults in Kenya. Finally, sex- and gender-disaggregated burden estimates were not produced.

## Conclusion

Among households with children aged <5 years across urban, peri-urban, and rural Kenya, the HUS showed a U-shaped distribution of HUS-defined SARI, with similarly high annual episode rates among young children and adults aged ≥65 years. This directly observed pattern persisted after national extrapolation. The analysis also identified two mechanisms of under-ascertainment by sentinel hospital surveillance: severe episodes for which hospitalization was recommended but did not occur and actual admissions to non-sentinel hospitals. Influenza-associated estimates showed a similar age pattern but require additional assumptions, particularly the application of all-age site/regional influenza positivity across age groups. These findings provide a community-based baseline for respiratory burden and vaccination policy analyses in Kenya.

## Declaration of competing interest

The authors have no competing interests to declare.

## Funding

The 2018 health utilization survey was supported by the US Centers for Disease Control and Prevention, which provided financial and technical support for survey implementation. The burden analysis and interpretation reported in this manuscript were conducted by the authors. The Centers for Disease Control and Prevention did not participate in the preparation of or the decision to submit the present manuscript.

## Ethical approval

This study was approved by the Kenya Medical Research Institute (KEMRI) Scientific & Ethics Review Unit (#3667), Washington State University Institutional Review Board (#16655), and the US Centers for Disease Control and Prevention (Protocol #7130). Written informed consent was obtained from adult participants and from parents or guardians for minors; assent was obtained from participants aged 13-17 years as applicable.

## Disclaimer

The findings and conclusions in this manuscript are those of the authors and do not necessarily represent the official position of the US Centers for Disease Control and Prevention or the authors’ institutions.

## Data availability statement

The data underlying this study are available from the corresponding author upon reasonable request in accordance with institutional data governance policies.

## Declaration of generative AI and AI-assisted technologies in the writing process

During the preparation of this work, the author used Claude (Anthropic) for editorial assistance, including language review and structural editing. After using this tool, the authors reviewed and edited the content as needed and take full responsibility for the content of the published article.

## CRediT authorship contribution statement

**Eric Osoro:** Conceptualization, Formal analysis, Investigation, Methodology, Project administration, Writing – original draft, Writing – review & editing. **Jeanette Dawa:** Methodology, Writing – review & editing. **Isaac Ngere:** Conceptualization, Investigation, Supervision, Writing – review & editing. **Nancy A. Otieno:** Investigation, Supervision, Writing – review & editing. **Godfrey Bigogo:** Conceptualization, Supervision, Writing – review & editing. **Mutono Nyamai:** Formal analysis, Writing – review & editing. **Samuel Waiguru:** Data curation, Formal analysis, Writing – review & editing. **M. Kariuki Njenga:** Conceptualization, Funding acquisition, Investigation, Supervision, Writing – review & editing. **Bryan O. Nyawanda:** Conceptualization, Data curation, Investigation, Methodology, Writing – review & editing.

## Appendix Supplementary materials

Image, application 1
