# Supplementary material for: Comparable burden of severe acute respiratory illness among children and older adults in Kenya: evidence from a multi-site health utilization survey
Source: IJID Reg. 2026 Sep 13;21:101011. doi: 10.1016/j.ijregi.2026.101011 (PMC13641029; doi:10.1016/j.ijregi.2026.101011)
Supplement: Supplementary file 1 [file mmc1.docx]

**Supplementary Figure 1. HUS-defined severe acute respiratory illness (SARI) episode rates by county, Kenya, 2018.** (A) Total HUS-defined SARI. (B) SARI with actual admission or recommendation for hospitalization. Choropleth maps show county-level point estimates per 100,000 population.

**Supplemental Table 1. Questions used to assess severity of reported pneumonia among persons aged ≥5 years**

| **#** | **Question** | **Responses** |
| --- | --- | --- |
| 1. | Was (NAME) limited in doing things that take a lot of energy (e.g. playing soccer, running, riding a bike) due to the illness? *(5-17 years)* | 1. Limited a lot 2. Limited a little 3. Not limited |
| 2. | Was (NAME) limited in taking care of him/herself (e.g. eating, dressing, bathing, or going to the toilet) due to the illness? *(≥5 years)* | 1. Limited a lot 2. Limited a little 3. Not limited |
| 3. | Was (NAME) limited in doing vigorous activities (e.g. running, lifting heavy objects, and digging, pushing a handcart) due to the illness? *(≥18 years)* | 1. Limited a lot 2. Limited a little 3. Not limited |
| 4. | Was (NAME) limited in lifting or carrying objects (e.g. basket of groceries/cereals, bucket of water) due to the illness? *(≥18 years)* | 1. Limited a lot 2. Limited a little 3. Not limited |
| 5. | Did (NAME) have difficulty performing the work or other activities (e.g. it took extra effort) as a result of the illness? *(≥18 years)* | 1. Limited a lot 2. Limited a little 3. Not limited |
| 6. | During the illness, were you/spouse LIMITED in the amount of time YOU had for your own needs because of your (NAME’s) physical health? *(<18 years)* | 1. Limited a lot 2. Limited a little 3. Not limited |

**Supplemental Table 2. Lower respiratory infection disability-adjusted life-year rates by county, Kenya, GBD 2016**

| **County** | **LRI DALY rate per 100,000 population (95% UI)** |
| --- | --- |
| Baringo | 2085 (1417–2833) |
| Bomet | 1935 (1290–2682) |
| Bungoma | 2413 (1711–3179) |
| Busia | 3625 (2633–4766) |
| Elgeyo-Marakwet | 2114 (1548–2806) |
| Embu | 2889 (2109–3848) |
| Garissa | 2883 (1968–4300) |
| Homabay | 4649 (3520–5830) |
| Isiolo | 3164 (2322–4148) |
| Kajiado | 2269 (1684–2924) |
| Kakamega | 3454 (2604–4490) |
| Kericho | 2042 (1390–2782) |
| Kiambu | 2388 (1783–3004) |
| Kilifi | 2708 (2037–3492) |
| Kirinyaga | 2722 (1985–3580) |
| Kisii | 2250 (1547–3059) |
| Kisumu | 3526 (2394–4848) |
| Kitui | 1918 (1328–2618) |
| Kwale | 2643 (1921–3495) |
| Laikipia | 1699 (1179–2384) |
| Lamu | 3752 (2883–4857) |
| Machakos | 2209 (1693–2805) |
| Makueni | 2239 (1548–3036) |
| Mandera | 3258 (1958–5075) |
| Marsabit | 2490 (1717–3461) |
| Meru | 3482 (2656–4374) |
| Migori | 4366 (3331–5557) |
| Mombasa | 3011 (2245–3903) |
| Muranga | 2655 (2039–3359) |
| Nairobi | 2024 (1552–2563) |
| Nakuru | 3446 (2479–4594) |
| Nandi | 1912 (1356–2570) |
| Narok | 2235 (1618–2941) |
| Nyamira | 2620 (1904–3413) |
| Nyandarua | 3670 (2820–4655) |
| Nyeri | 3326 (2644–4037) |
| Samburu | 2346 (1559–3305) |
| Siaya | 3508 (2429–4774) |
| Taita taveta | 3390 (2669–4230) |
| Tana River | 3840 (2758–5118) |
| Tharaka-nithi | 2880 (2187–3652) |
| Trans Nzoia | 1946 (1330–2642) |
| Turkana | 2803 (1964–3974) |
| Uasin Gishu | 2219 (1612–2923) |
| Vihiga | 3739 (2501–5404) |
| Wajir | 2892 (1834–4627) |
| West Pokot | 1761 (1143–2527) |
| DALY, disability-adjusted life year; LRI, lower respiratory infection; UI, uncertainty interval. County-specific modeled LRI DALY rates were used to construct the relative geographic scaling factors. | |

**Supplemental Table 3. Summary of data sources and their application in the burden model**

| **Data source** | **Key variables used** | **Coverage** | **Application in burden model** |
| --- | --- | --- | --- |
| Health Utilization Survey (HUS), 2018 | Age-specific HUS-defined SARI episode rates; proportion of HUS-defined SARI episodes with actual admission or recommendation for hospitalization | Kakamega, Nakuru and Siaya counties | Pooled age-specific HUS-defined SARI episode rates and admission-or-recommendation proportions used as base inputs for county extrapolation |
| Influenza sentinel surveillance, 2017–2019 | Influenza test positivity among SARI surveillance samples | Seven surveillance sites: Kakamega, Kenyatta, Marsabit, Mombasa, Nakuru, Nyeri and Siaya | Influenza attribution using surveillance-site positivity and predefined geographic proxy mapping; pooled Kakamega-Nakuru-Siaya and Eastern proxy inputs were also used |
| Global Burden of Disease Study, 2016 | County-level YLL and YLD estimates for lower respiratory infections | All 47 counties | Relative county geographic adjustment based on the LRI DALY rate divided by the pooled Kakamega-Nakuru-Siaya reference rate |
| Kenya Demographic and Health Survey, 2014 | Weighted proportion of children aged <5 years with ARI for whom care was sought | National and county-level domains | Relative geographic healthcare-seeking modifier; county probability divided by the pooled Kakamega-Nakuru-Siaya probability and applied across age groups |
| Kenya population projection derived from the 2009 Population and Housing Census | Projected 2018 county and age-group population | All 47 counties and national total | Conversion of estimated rates to annual numbers of SARI episodes |
| Abbreviations: ARI, acute respiratory illness; DALY, disability-adjusted life year; HUS, health utilization survey; KDHS, Kenya Demographic and Health Survey; LRI, lower respiratory infection; SARI, severe acute respiratory illness; YLD, years lived with disability; YLL, years of life lost. | | | |

**Supplemental Table 4. Summary of HUS-defined SARI findings in the three base counties, Kenya, 2018**

| **Base county** | **Age group** | **Analytic participants** | **All SARI episodes** | **SARI episodes with actual admission or recommendation for hospitalization** | **Actual admissions** | **Recommendation for hospitalization without actual admission** | **SARI episodes without admission/recommendation** | **All SARI episode rate per 100,000 (95% UI)** | **Admission-or-recommendation SARI episode rate per 100,000 (95% UI)** | **No admission/recommendation SARI episode rate per 100,000 (95% UI)** |
| --- | --- | --- | --- | --- | --- | --- | --- | --- | --- | --- |
| Kakamega | <2yrs | 550 | 40 | 14 | 12 | 2 | 26 | 7273 (3925–11567) | 2545 (786–4762) | 4727 (2075–8561) |
| Kakamega | 2-4yrs | 1119 | 81 | 35 | 27 | 8 | 46 | 7239 (4627–10403) | 3128 (1509–4962) | 4111 (2137–6927) |
| Kakamega | 5-14yrs | 2006 | 64 | 19 | 14 | 5 | 45 | 3190 (1862–4847) | 947 (421–1581) | 2243 (1043–3830) |
| Kakamega | 15-49yrs | 3185 | 83 | 15 | 6 | 9 | 68 | 2606 (1608–3833) | 471 (138–919) | 2135 (1279–3236) |
| Kakamega | 50-64yrs | 371 | 16 | 7 | 5 | 2 | 9 | 4313 (1128–8743) | 1887 (0–5163) | 2426 (337–5294) |
| Kakamega | >=65yrs | 152 | 17 | 7 | 5 | 2 | 10 | 11184 (3311–23405) | 4605 (0–14815) | 6579 (1613–13386) |
| Kakamega | Total | 7383 | 301 | 97 | 69 | 28 | 204 | 4077 (3050–5349) | 1314 (858–1816) | 2763 (1931–3846) |
| Nakuru | <2yrs | 555 | 88 | 28 | 24 | 4 | 60 | 15856 (9851–23363) | 5045 (1996–9291) | 10811 (5641–17768) |
| Nakuru | 2-4yrs | 907 | 149 | 31 | 26 | 5 | 118 | 16428 (11255–23193) | 3418 (1597–5626) | 13010 (8356–19215) |
| Nakuru | 5-14yrs | 1492 | 30 | 8 | 7 | 1 | 22 | 2011 (749–3630) | 536 (0–1280) | 1475 (518–2646) |
| Nakuru | 15-49yrs | 2942 | 71 | 28 | 15 | 13 | 43 | 2413 (1364–3376) | 952 (405–1440) | 1462 (737–2237) |
| Nakuru | 50-64yrs | 278 | 14 | 3 | 1 | 2 | 11 | 5036 (1293–9402) | 1079 (0–4016) | 3957 (934–7725) |
| Nakuru | >=65yrs | 80 | 5 | 1 | 1 | 0 | 4 | 6250 (0–17144) | 1250 (0–6557) | 5000 (0–14707) |
| Nakuru | Total | 6254 | 357 | 99 | 74 | 25 | 258 | 5708 (4217–7427) | 1583 (949–2201) | 4125 (2874–5660) |
| Siaya | <2yrs | 508 | 31 | 7 | 6 | 1 | 24 | 6102 (2202–12468) | 1378 (217–2868) | 4724 (1202–10772) |
| Siaya | 2-4yrs | 1156 | 89 | 22 | 19 | 3 | 67 | 7699 (5109–10925) | 1903 (804–3267) | 5796 (3552–8704) |
| Siaya | 5-14yrs | 2270 | 25 | 7 | 5 | 2 | 18 | 1101 (518–1816) | 308 (37–753) | 793 (315–1405) |
| Siaya | 15-49yrs | 3059 | 62 | 19 | 17 | 2 | 43 | 2027 (1293–2924) | 621 (237–1174) | 1406 (836–2090) |
| Siaya | 50-64yrs | 327 | 13 | 9 | 9 | 0 | 4 | 3976 (935–8383) | 2752 (283–6785) | 1223 (0–3170) |
| Siaya | >=65yrs | 131 | 11 | 2 | 1 | 1 | 9 | 8397 (934–18812) | 1527 (0–4965) | 6870 (699–16949) |
| Siaya | Total | 7451 | 231 | 66 | 57 | 9 | 165 | 3100 (2307–4060) | 886 (541–1308) | 2214 (1608–2966) |
| Combined | <2yrs | 1613 | 159 | 49 | 42 | 7 | 110 | 9857 (6842–13305) | 3038 (1702–4600) | 6820 (4215–9922) |
| Combined | 2-4yrs | 3182 | 319 | 88 | 72 | 16 | 231 | 10025 (7996–12432) | 2766 (1851–3770) | 7260 (5475–9396) |
| Combined | 5-14yrs | 5768 | 119 | 34 | 26 | 8 | 85 | 2063 (1430–2810) | 589 (309–921) | 1474 (936–2116) |
| Combined | 15-49yrs | 9186 | 216 | 62 | 38 | 24 | 154 | 2351 (1809–2921) | 675 (410–968) | 1676 (1246–2152) |
| Combined | 50-64yrs | 976 | 43 | 19 | 15 | 4 | 24 | 4406 (2328–6843) | 1947 (550–3803) | 2459 (1135–4049) |
| Combined | >=65yrs | 363 | 33 | 10 | 7 | 3 | 23 | 9091 (4132–15551) | 2755 (292–7165) | 6336 (2550–11194) |
| Combined | Total | 21088 | 889 | 262 | 200 | 62 | 627 | 4216 (3528–4980) | 1242 (952–1537) | 2973 (2412–3606) |
| Combined is the aggregate of Kakamega, Nakuru and Siaya. Admission-or-recommendation includes actual admissions and recommendation-only episodes. No admission/recommendation denotes episodes with neither actual admission nor recommendation for hospitalization. HUS, health utilization survey; SARI, severe acute respiratory illness; UI, uncertainty interval. | | | | | | | | | | |

**Supplemental Table 5A. HUS-defined SARI episode rates per 100,000 population at national and county level by age group, Kenya, 2018**

| **National/County** | **<2 years** | **2–4 years** | **5–14 years** | **15–49 years** | **50–64 years** | **≥65 years** | **All age groups** |
| --- | --- | --- | --- | --- | --- | --- | --- |
| KENYA | 7758 (5008–11156) | 7890 (5698–10629) | 1624 (1040–2354) | 1851 (1302–2493) | 3467 (1746–5651) | 7155 (3108–12682) | 3318 (2477–4326) |
| Baringo | 5939 (3554–9140) | 6040 (3961–8717) | 1243 (744–1910) | 1417 (910–2068) | 2654 (1264–4484) | 5477 (2288–10117) | 2540 (1706–3582) |
| Bomet | 5510 (3283–8445) | 5604 (3599–8226) | 1153 (679–1802) | 1314 (832–1946) | 2463 (1181–4241) | 5081 (2121–9531) | 2356 (1560–3364) |
| Bungoma | 6872 (4205–10397) | 6989 (4712–10033) | 1438 (876–2195) | 1639 (1084–2344) | 3071 (1493–5281) | 6337 (2667–11531) | 2939 (2031–4107) |
| Busia | 10324 (6373–15620) | 10500 (7139–15072) | 2161 (1334–3306) | 2463 (1634–3538) | 4614 (2251–7830) | 9521 (4044–17437) | 4415 (3080–6122) |
| Elgeyo-Marakwet | 6022 (3731–9122) | 6124 (4182–8766) | 1260 (778–1916) | 1436 (955–2061) | 2691 (1319–4561) | 5553 (2385–10241) | 2575 (1802–3610) |
| Embu | 8226 (5119–12434) | 8366 (5675–11988) | 1722 (1061–2629) | 1962 (1299–2826) | 3677 (1784–6243) | 7587 (3193–14060) | 3518 (2454–4918) |
| Garissa | 8210 (4888–13148) | 8350 (5413–12739) | 1718 (1015–2770) | 1958 (1250–2980) | 3670 (1751–6555) | 7572 (3084–14377) | 3511 (2327–5225) |
| Homabay | 13239 (8449–19660) | 13464 (9371–18708) | 2771 (1739–4131) | 3158 (2169–4436) | 5917 (2929–9909) | 12210 (5266–22147) | 5662 (4074–7696) |
| Isiolo | 9010 (5616–13545) | 9164 (6316–13008) | 1886 (1165–2861) | 2149 (1434–3055) | 4027 (1986–6893) | 8310 (3548–15220) | 3853 (2705–5327) |
| Kajiado | 6463 (4002–9639) | 6573 (4558–9212) | 1353 (835–2030) | 1542 (1036–2176) | 2889 (1427–4846) | 5960 (2547–10877) | 2764 (1956–3787) |
| Kakamega | 7258 (3831–12018) | 7224 (4408–10765) | 3184 (1789–5007) | 2601 (1553–3990) | 4304 (1116–8948) | 11162 (3242–23977) | 4069 (2853–5616) |
| Kericho | 5815 (3480–8921) | 5913 (3845–8583) | 1217 (728–1890) | 1387 (882–2033) | 2599 (1234–4448) | 5362 (2252–9938) | 2487 (1652–3532) |
| Kiambu | 6799 (4253–10051) | 6915 (4770–9600) | 1423 (893–2115) | 1622 (1098–2252) | 3039 (1492–5033) | 6271 (2659–11309) | 2908 (2076–3913) |
| Kilifi | 7713 (4807–11495) | 7844 (5470–11029) | 1614 (1013–2418) | 1840 (1252–2615) | 3447 (1703–5784) | 7113 (3071–13130) | 3298 (2349–4512) |
| Kirinyaga | 7752 (4797–11638) | 7884 (5393–11172) | 1622 (1000–2451) | 1849 (1231–2633) | 3465 (1717–5821) | 7149 (3032–13007) | 3315 (2319–4556) |
| Kisii | 6408 (3895–9809) | 6517 (4277–9424) | 1341 (802–2068) | 1529 (989–2234) | 2864 (1381–4893) | 5910 (2480–10884) | 2741 (1854–3878) |
| Kisumu | 10043 (5969–15445) | 10214 (6609–14946) | 2102 (1244–3276) | 2396 (1513–3510) | 4489 (2139–7775) | 9262 (3811–17096) | 4295 (2847–6132) |
| Kitui | 5461 (3311–8352) | 5554 (3652–8027) | 1143 (681–1769) | 1303 (843–1894) | 2441 (1160–4155) | 5036 (2112–9299) | 2335 (1580–3297) |
| Kwale | 7527 (4658–11393) | 7655 (5201–10924) | 1575 (984–2376) | 1795 (1195–2579) | 3364 (1636–5620) | 6941 (2918–12692) | 3219 (2258–4476) |
| Laikipia | 4838 (2905–7456) | 4920 (3254–7188) | 1013 (606–1583) | 1154 (748–1704) | 2162 (1040–3719) | 4462 (1877–8391) | 2069 (1398–2968) |
| Lamu | 10686 (6718–15883) | 10868 (7597–15248) | 2237 (1396–3376) | 2549 (1730–3598) | 4776 (2375–7971) | 9855 (4290–17813) | 4570 (3278–6251) |
| Machakos | 6291 (3993–9362) | 6398 (4478–8915) | 1317 (835–1987) | 1501 (1022–2113) | 2812 (1395–4664) | 5802 (2514–10427) | 2690 (1946–3659) |
| Makueni | 6377 (3859–9698) | 6486 (4306–9411) | 1335 (808–2073) | 1521 (992–2226) | 2850 (1390–4898) | 5881 (2494–10966) | 2727 (1852–3848) |
| Mandera | 9280 (5162–15263) | 9438 (5636–14859) | 1942 (1085–3233) | 2214 (1316–3483) | 4148 (1926–7531) | 8558 (3492–16571) | 3969 (2423–6088) |
| Marsabit | 7090 (4257–10997) | 7211 (4741–10593) | 1484 (896–2320) | 1691 (1084–2486) | 3169 (1505–5478) | 6539 (2738–12220) | 3032 (2032–4358) |
| Meru | 9915 (6256–14622) | 10084 (7065–13969) | 2075 (1316–3072) | 2365 (1621–3283) | 4431 (2205–7385) | 9144 (3978–16354) | 4240 (3041–5696) |
| Migori | 12435 (7840–18387) | 12646 (8818–17591) | 2602 (1619–3900) | 2966 (2014–4160) | 5558 (2736–9300) | 11468 (4987–20726) | 5318 (3815–7198) |
| Mombasa | 8576 (5392–12718) | 8722 (6033–12311) | 1795 (1111–2696) | 2046 (1371–2895) | 3833 (1887–6421) | 7909 (3354–14361) | 3667 (2600–5032) |
| Muranga | 7562 (4758–11117) | 7690 (5366–10670) | 1583 (1002–2366) | 1804 (1240–2515) | 3380 (1678–5610) | 6974 (2978–12535) | 3234 (2331–4369) |
| Nairobi | 5765 (3653–8508) | 5863 (4130–8164) | 1207 (766–1795) | 1375 (943–1917) | 2577 (1298–4322) | 5317 (2318–9509) | 2465 (1783–3344) |
| Nakuru | 15785 (9446–24439) | 16355 (10556–24100) | 2002 (725–3679) | 2403 (1315–3514) | 5014 (1267–9500) | 6222 (0–17079) | 5683 (3914–7821) |
| Nandi | 5445 (3345–8239) | 5538 (3752–7917) | 1140 (694–1755) | 1299 (854–1873) | 2434 (1176–4122) | 5022 (2131–9271) | 2329 (1614–3253) |
| Narok | 6366 (3939–9593) | 6474 (4440–9164) | 1332 (825–2029) | 1518 (1020–2155) | 2845 (1401–4820) | 5871 (2503–10724) | 2722 (1911–3763) |
| Nyamira | 7462 (4666–11167) | 7589 (5171–10719) | 1562 (967–2345) | 1780 (1187–2520) | 3335 (1627–5627) | 6882 (2963–12527) | 3191 (2222–4406) |
| Nyandarua | 10452 (6581–15317) | 10630 (7389–14721) | 2188 (1384–3255) | 2493 (1693–3474) | 4672 (2318–7763) | 9640 (4166–17493) | 4470 (3198–6018) |
| Nyeri | 9474 (6054–13790) | 9635 (6821–13186) | 1983 (1272–2902) | 2260 (1576–3098) | 4234 (2127–6960) | 8737 (3776–15507) | 4052 (2982–5387) |
| Samburu | 6680 (3968–10551) | 6794 (4386–10101) | 1398 (832–2207) | 1593 (1013–2368) | 2986 (1427–5205) | 6161 (2556–11547) | 2857 (1879–4140) |
| Siaya | 6184 (2143–13324) | 7802 (4744–12083) | 1116 (499–1950) | 2054 (1204–3167) | 4029 (916–8911) | 8509 (972–20076) | 3142 (2080–4533) |
| Taita taveta | 9654 (6150–14224) | 9818 (6989–13606) | 2021 (1301–3004) | 2303 (1591–3192) | 4315 (2168–7156) | 8903 (3868–15971) | 4129 (3019–5556) |
| Tana River | 10937 (6696–16709) | 11123 (7487–15870) | 2289 (1413–3484) | 2609 (1731–3745) | 4888 (2378–8330) | 10087 (4323–18387) | 4677 (3233–6517) |
| Tharaka-nithi | 8202 (5185–12183) | 8341 (5806–11635) | 1717 (1078–2567) | 1956 (1328–2752) | 3666 (1807–6164) | 7564 (3240–13620) | 3508 (2505–4796) |
| Trans Nzoia | 5543 (3339–8522) | 5638 (3689–8174) | 1160 (698–1795) | 1322 (853–1922) | 2478 (1186–4245) | 5112 (2119–9589) | 2371 (1595–3352) |
| Turkana | 7984 (4840–12501) | 8120 (5378–12025) | 1671 (1009–2632) | 1904 (1242–2828) | 3568 (1726–6230) | 7363 (3138–13821) | 3414 (2311–4960) |
| Uasin Gishu | 6320 (3887–9465) | 6428 (4365–9102) | 1323 (818–2016) | 1508 (1006–2152) | 2825 (1373–4755) | 5829 (2465–10800) | 2703 (1878–3711) |
| Vihiga | 10648 (6272–16769) | 10829 (6950–16190) | 2229 (1309–3540) | 2540 (1590–3831) | 4759 (2265–8346) | 9820 (4094–18945) | 4554 (2989–6681) |
| Wajir | 8237 (4726–13760) | 8377 (5208–13345) | 1724 (978–2901) | 1965 (1187–3151) | 3681 (1713–6784) | 7596 (3112–14954) | 3523 (2235–5516) |
| West Pokot | 5016 (2914–7933) | 5101 (3216–7695) | 1050 (612–1665) | 1197 (745–1804) | 2242 (1057–3882) | 4626 (1927–8775) | 2145 (1385–3144) |

**Supplemental Table 5B. SARI episode rates with actual admission or recommendation for hospitalization per 100,000 population at national and county level by age group, Kenya, 2018**

| **National/County** | **<2 years** | **2–4 years** | **5–14 years** | **15–49 years** | **50–64 years** | **≥65 years** | **All age groups** |
| --- | --- | --- | --- | --- | --- | --- | --- |
| KENYA | 2322 (1225–3771) | 2114 (1305–3150) | 450 (226–744) | 516 (292–798) | 1488 (411–3048) | 2105 (221–5667) | 950 (653–1323) |
| Baringo | 1807 (871–3193) | 1645 (920–2679) | 351 (162–633) | 402 (207–675) | 1158 (302–2509) | 1639 (173–4607) | 739 (443–1145) |
| Bomet | 1597 (708–2885) | 1454 (726–2501) | 310 (132–580) | 355 (166–627) | 1024 (259–2328) | 1448 (153–4066) | 653 (351–1065) |
| Bungoma | 1539 (716–2756) | 1401 (740–2326) | 299 (135–544) | 342 (171–592) | 986 (254–2178) | 1396 (144–3892) | 629 (363–997) |
| Busia | 3936 (1964–6803) | 3583 (2078–5711) | 764 (366–1334) | 875 (470–1459) | 2522 (676–5366) | 3570 (380–9768) | 1610 (1017–2439) |
| Elgeyo-Marakwet | 2564 (1303–4436) | 2334 (1365–3704) | 498 (240–876) | 570 (305–934) | 1643 (453–3477) | 2325 (246–6364) | 1049 (669–1573) |
| Embu | 3237 (1605–5635) | 2947 (1680–4805) | 628 (296–1133) | 719 (379–1210) | 2074 (560–4448) | 2936 (318–8163) | 1324 (823–2061) |
| Garissa | 1283 (0–3094) | 1168 (0–2718) | 249 (0–615) | 285 (0–673) | 822 (0–2386) | 1164 (0–4071) | 525 (0–1187) |
| Homabay | 4089 (2086–6904) | 3722 (2182–5795) | 793 (392–1364) | 908 (496–1471) | 2620 (712–5551) | 3708 (392–10097) | 1672 (1080–2451) |
| Isiolo | 2600 (1239–4579) | 2367 (1276–3896) | 504 (230–919) | 578 (292–984) | 1666 (432–3601) | 2358 (254–6536) | 1063 (624–1662) |
| Kajiado | 1831 (884–3178) | 1667 (940–2668) | 355 (167–631) | 407 (213–685) | 1173 (308–2506) | 1660 (178–4577) | 749 (455–1138) |
| Kakamega | 2120 (634–4215) | 2604 (1177–4451) | 789 (337–1406) | 392 (110–802) | 1571 (0–4476) | 3835 (0–12484) | 1094 (660–1672) |
| Kericho | 1736 (822–3156) | 1580 (848–2634) | 337 (155–622) | 386 (196–668) | 1112 (291–2463) | 1574 (167–4459) | 710 (416–1136) |
| Kiambu | 2338 (1158–4064) | 2129 (1205–3424) | 454 (214–797) | 519 (279–865) | 1498 (394–3159) | 2120 (227–5744) | 956 (592–1449) |
| Kilifi | 1994 (958–3487) | 1815 (1000–2974) | 387 (180–688) | 443 (229–744) | 1278 (336–2793) | 1808 (187–5045) | 816 (488–1252) |
| Kirinyaga | 1198 (0–3597) | 1090 (0–3241) | 232 (0–715) | 266 (0–784) | 767 (0–2598) | 1086 (0–4347) | 490 (0–1402) |
| Kisii | 1869 (857–3383) | 1701 (870–2931) | 363 (157–682) | 415 (199–735) | 1197 (300–2668) | 1695 (178–4829) | 764 (422–1246) |
| Kisumu | 3510 (1631–6272) | 3196 (1713–5406) | 681 (311–1251) | 780 (387–1374) | 2249 (580–5038) | 3183 (333–8843) | 1436 (825–2299) |
| Kitui | 1425 (662–2583) | 1297 (679–2200) | 277 (124–515) | 317 (156–555) | 913 (235–2014) | 1292 (135–3664) | 583 (329–948) |
| Kwale | 2065 (968–3698) | 1880 (1010–3164) | 401 (181–735) | 459 (225–800) | 1323 (334–2934) | 1873 (199–5175) | 845 (484–1349) |
| Laikipia | 1606 (748–2903) | 1462 (779–2456) | 312 (142–580) | 357 (180–624) | 1029 (270–2273) | 1457 (155–4119) | 657 (380–1056) |
| Lamu | 3332 (1635–5755) | 3033 (1695–4903) | 646 (305–1157) | 740 (390–1234) | 2135 (563–4646) | 3021 (322–8246) | 1363 (828–2093) |
| Machakos | 1557 (729–2782) | 1417 (743–2359) | 302 (135–550) | 346 (170–600) | 998 (255–2165) | 1412 (150–3958) | 637 (359–1014) |
| Makueni | 1868 (869–3340) | 1701 (918–2854) | 363 (165–665) | 415 (208–718) | 1197 (319–2641) | 1694 (178–4840) | 764 (448–1222) |
| Mandera | 2458 (0–5202) | 2237 (0–4563) | 477 (0–1038) | 546 (0–1124) | 1575 (0–4036) | 2229 (0–6849) | 1005 (0–1953) |
| Marsabit | 2082 (836–3819) | 1895 (855–3289) | 404 (160–756) | 463 (197–824) | 1334 (310–3009) | 1888 (189–5349) | 851 (408–1402) |
| Meru | 4168 (2098–6947) | 3794 (2218–5922) | 809 (389–1383) | 926 (500–1487) | 2671 (704–5577) | 3780 (402–10280) | 1705 (1099–2487) |
| Migori | 4225 (2100–7185) | 3846 (2232–6107) | 820 (390–1444) | 939 (500–1545) | 2707 (708–5661) | 3831 (402–10328) | 1728 (1086–2582) |
| Mombasa | 2975 (1404–5156) | 2709 (1472–4392) | 577 (265–1019) | 661 (337–1101) | 1907 (505–4071) | 2698 (280–7403) | 1217 (719–1868) |
| Muranga | 2678 (1313–4596) | 2438 (1364–3901) | 520 (244–906) | 595 (309–970) | 1716 (455–3577) | 2428 (257–6576) | 1095 (675–1631) |
| Nairobi | 1709 (816–3027) | 1556 (855–2551) | 332 (154–595) | 380 (196–638) | 1095 (286–2398) | 1550 (162–4352) | 699 (415–1084) |
| Nakuru | 5641 (2130–10895) | 3822 (1659–6611) | 600 (0–1487) | 1064 (426–1711) | 1207 (0–4580) | 1398 (0–7071) | 1770 (977–2674) |
| Nandi | 1625 (797–2874) | 1480 (821–2426) | 315 (148–568) | 361 (185–619) | 1042 (273–2315) | 1474 (156–4133) | 665 (401–1042) |
| Narok | 1981 (965–3462) | 1803 (1020–2937) | 384 (181–688) | 440 (229–746) | 1269 (332–2757) | 1796 (191–4924) | 810 (495–1250) |
| Nyamira | 2168 (895–4525) | 1974 (926–3892) | 421 (168–885) | 482 (215–955) | 1389 (320–3459) | 1966 (203–6055) | 887 (442–1678) |
| Nyandarua | 2488 (888–5015) | 2265 (889–4355) | 483 (167–1007) | 553 (210–1096) | 1594 (343–3797) | 2256 (218–6884) | 1018 (423–1884) |
| Nyeri | 3140 (1494–5307) | 2858 (1593–4558) | 609 (284–1062) | 698 (363–1143) | 2012 (530–4272) | 2847 (296–7840) | 1284 (770–1931) |
| Samburu | 1677 (679–3206) | 1527 (689–2804) | 325 (127–645) | 373 (160–697) | 1075 (252–2549) | 1521 (157–4475) | 686 (327–1201) |
| Siaya | 1603 (259–3526) | 2214 (851–4184) | 359 (32–915) | 723 (261–1463) | 3202 (288–7970) | 1776 (0–5948) | 1030 (554–1700) |
| Taita taveta | 3506 (1791–5920) | 3192 (1880–5072) | 680 (329–1189) | 779 (421–1274) | 2247 (610–4734) | 3179 (341–8671) | 1434 (924–2143) |
| Tana River | 3508 (1731–6132) | 3193 (1798–5195) | 681 (321–1215) | 779 (411–1312) | 2248 (594–4844) | 3181 (338–8730) | 1435 (877–2206) |
| Tharaka-nithi | 2501 (1217–4328) | 2277 (1267–3630) | 485 (228–856) | 556 (292–931) | 1603 (429–3438) | 2268 (239–6222) | 1023 (621–1557) |
| Trans Nzoia | 1974 (943–3439) | 1797 (981–2906) | 383 (173–679) | 439 (222–741) | 1265 (328–2702) | 1790 (184–4885) | 807 (473–1239) |
| Turkana | 2300 (1007–4329) | 2094 (1045–3738) | 446 (190–864) | 511 (245–941) | 1474 (370–3417) | 2086 (215–5968) | 941 (505–1597) |
| Uasin Gishu | 1528 (680–2703) | 1391 (721–2323) | 297 (128–536) | 340 (167–584) | 979 (246–2118) | 1386 (141–3893) | 625 (346–986) |
| Vihiga | 3023 (1433–5560) | 2752 (1487–4761) | 587 (268–1106) | 672 (340–1193) | 1937 (511–4429) | 2741 (289–7867) | 1236 (722–2036) |
| Wajir | 1257 (421–2869) | 1144 (407–2491) | 244 (78–563) | 279 (95–621) | 806 (167–2191) | 1140 (112–3802) | 514 (190–1080) |
| West Pokot | 1766 (824–3195) | 1608 (873–2704) | 343 (156–630) | 392 (199–682) | 1132 (290–2519) | 1602 (171–4542) | 722 (422–1155) |

**Supplemental Table 5C. SARI without admission/recommendation episode rates per 100,000 population at national and county level by age group, Kenya, 2018**

| **National/County** | **<2 years** | **2–4 years** | **5–14 years** | **15–49 years** | **50–64 years** | **≥65 years** | **All age groups** |
| --- | --- | --- | --- | --- | --- | --- | --- |
| KENYA | 5436 (3195–8333) | 5776 (3988–8026) | 1173 (701–1782) | 1335 (906–1836) | 1980 (860–3352) | 5049 (1964–9198) | 2368 (1719–3169) |
| Baringo | 4132 (2226–6752) | 4395 (2755–6607) | 892 (487–1430) | 1015 (627–1508) | 1496 (591–2698) | 3838 (1422–7280) | 1801 (1177–2599) |
| Bomet | 3913 (2084–6444) | 4150 (2552–6348) | 843 (461–1389) | 959 (581–1462) | 1439 (558–2664) | 3633 (1324–7032) | 1703 (1087–2529) |
| Bungoma | 5333 (3070–8394) | 5587 (3651–8184) | 1140 (660–1785) | 1297 (835–1896) | 2085 (967–3608) | 4942 (2006–9054) | 2309 (1555–3288) |
| Busia | 6388 (3210–10679) | 6916 (4307–10435) | 1397 (737–2304) | 1588 (978–2361) | 2092 (467–4099) | 5952 (1758–11805) | 2805 (1854–4020) |
| Elgeyo-Marakwet | 3458 (1620–5891) | 3790 (2324–5766) | 763 (387–1270) | 867 (517–1316) | 1048 (30–2237) | 3228 (692–6669) | 1527 (992–2222) |
| Embu | 4989 (2420–8388) | 5419 (3290–8207) | 1094 (551–1808) | 1243 (739–1867) | 1602 (215–3215) | 4651 (1219–9356) | 2194 (1414–3177) |
| Garissa | 6927 (3884–11593) | 7182 (4466–11271) | 1469 (822–2434) | 1673 (1029–2625) | 2847 (1267–5335) | 6408 (2520–12486) | 2986 (1905–4587) |
| Homabay | 9150 (5148–14535) | 9742 (6474–14122) | 1977 (1144–3117) | 2250 (1469–3239) | 3297 (1315–5833) | 8502 (3168–15859) | 3990 (2779–5550) |
| Isiolo | 6411 (3537–10280) | 6797 (4418–10006) | 1381 (782–2191) | 1572 (1008–2298) | 2361 (979–4242) | 5952 (2281–11135) | 2790 (1887–3969) |
| Kajiado | 4632 (2598–7312) | 4906 (3242–7167) | 997 (572–1575) | 1135 (740–1649) | 1715 (714–3023) | 4300 (1695–8022) | 2015 (1377–2844) |
| Kakamega | 5139 (2339–9184) | 4620 (2452–7713) | 2395 (1164–4097) | 2209 (1277–3440) | 2733 (589–5739) | 7328 (2025–14839) | 2975 (1981–4272) |
| Kericho | 4079 (2156–6633) | 4333 (2674–6502) | 880 (478–1431) | 1001 (613–1496) | 1486 (580–2666) | 3788 (1397–7280) | 1777 (1139–2588) |
| Kiambu | 4461 (2333–7166) | 4787 (3050–6936) | 969 (524–1540) | 1102 (686–1586) | 1541 (459–2806) | 4150 (1365–7877) | 1952 (1297–2720) |
| Kilifi | 5719 (3273–8951) | 6028 (4049–8693) | 1227 (717–1911) | 1397 (919–2020) | 2169 (949–3756) | 5305 (2104–9799) | 2483 (1716–3473) |
| Kirinyaga | 6554 (3393–10248) | 6793 (3994–9943) | 1390 (736–2184) | 1583 (924–2331) | 2697 (955–4785) | 6063 (2246–11253) | 2825 (1688–4050) |
| Kisii | 4539 (2459–7431) | 4816 (3003–7202) | 979 (533–1582) | 1113 (687–1665) | 1667 (637–3030) | 4215 (1525–8051) | 1976 (1277–2874) |
| Kisumu | 6533 (3253–10949) | 7019 (4201–10731) | 1421 (737–2362) | 1616 (955–2469) | 2239 (608–4321) | 6079 (1941–12044) | 2860 (1785–4253) |
| Kitui | 4036 (2220–6424) | 4256 (2699–6291) | 866 (483–1389) | 986 (618–1456) | 1527 (650–2709) | 3744 (1458–7028) | 1753 (1144–2515) |
| Kwale | 5462 (3050–8725) | 5775 (3765–8485) | 1175 (673–1856) | 1337 (854–1961) | 2041 (832–3611) | 5069 (1902–9430) | 2374 (1599–3376) |
| Laikipia | 3232 (1656–5395) | 3458 (2148–5309) | 701 (378–1150) | 797 (489–1219) | 1133 (355–2165) | 3005 (998–5949) | 1412 (912–2097) |
| Lamu | 7355 (4085–11791) | 7835 (5163–11439) | 1590 (899–2554) | 1809 (1173–2641) | 2641 (999–4772) | 6834 (2529–12907) | 3208 (2207–4519) |
| Machakos | 4734 (2756–7420) | 4981 (3344–7196) | 1015 (599–1583) | 1155 (765–1664) | 1814 (809–3149) | 4390 (1761–7984) | 2054 (1433–2865) |
| Makueni | 4509 (2438–7295) | 4785 (3028–7197) | 972 (539–1579) | 1106 (688–1658) | 1653 (651–2997) | 4187 (1574–8003) | 1963 (1282–2856) |
| Mandera | 6822 (3328–12131) | 7201 (4036–11980) | 1465 (748–2592) | 1668 (931–2749) | 2573 (825–5182) | 6330 (2190–12792) | 2964 (1710–4807) |
| Marsabit | 5008 (2666–8411) | 5315 (3289–8190) | 1080 (598–1792) | 1229 (754–1903) | 1835 (689–3478) | 4651 (1726–9139) | 2181 (1400–3280) |
| Meru | 5747 (2787–9609) | 6289 (3929–9345) | 1266 (652–2068) | 1439 (888–2116) | 1761 (142–3653) | 5364 (1231–10824) | 2536 (1689–3606) |
| Migori | 8210 (4449–13206) | 8800 (5762–12823) | 1783 (992–2856) | 2028 (1299–2942) | 2850 (941–5283) | 7637 (2654–14555) | 3590 (2456–5026) |
| Mombasa | 5600 (2935–9128) | 6013 (3868–8997) | 1218 (660–1971) | 1385 (863–2041) | 1926 (532–3698) | 5211 (1675–10101) | 2451 (1631–3518) |
| Muranga | 4884 (2580–7935) | 5253 (3376–7694) | 1063 (577–1706) | 1209 (768–1769) | 1664 (485–3128) | 4545 (1504–8706) | 2139 (1451–3023) |
| Nairobi | 4056 (2268–6465) | 4308 (2867–6209) | 875 (499–1369) | 996 (648–1431) | 1482 (583–2620) | 3767 (1418–6933) | 1767 (1222–2471) |
| Nakuru | 10144 (4623–17818) | 12533 (7510–19468) | 1402 (459–2611) | 1338 (603–2187) | 3807 (753–7792) | 4824 (0–14670) | 3913 (2495–5741) |
| Nandi | 3820 (2093–6122) | 4058 (2586–6020) | 824 (458–1318) | 938 (592–1379) | 1392 (542–2507) | 3548 (1302–6637) | 1664 (1109–2380) |
| Narok | 4385 (2369–7014) | 4671 (3050–6848) | 948 (536–1514) | 1078 (686–1574) | 1576 (592–2821) | 4074 (1495–7716) | 1912 (1287–2721) |
| Nyamira | 5294 (2632–8472) | 5615 (3359–8278) | 1141 (588–1818) | 1298 (765–1905) | 1946 (453–3509) | 4916 (1602–9166) | 2305 (1412–3291) |
| Nyandarua | 7964 (4349–12448) | 8365 (5378–12063) | 1705 (958–2638) | 1941 (1223–2793) | 3077 (1223–5431) | 7383 (2856–13514) | 3452 (2277–4831) |
| Nyeri | 6334 (3474–10019) | 6777 (4472–9766) | 1374 (781–2134) | 1562 (1020–2250) | 2222 (692–4026) | 5890 (2050–10987) | 2767 (1915–3828) |
| Samburu | 5003 (2700–8305) | 5267 (3228–8129) | 1073 (593–1755) | 1221 (743–1864) | 1911 (785–3498) | 4640 (1777–8872) | 2171 (1373–3258) |
| Siaya | 4581 (948–11199) | 5588 (3056–9227) | 757 (244–1463) | 1331 (692–2177) | 827 (0–3080) | 6733 (423–17454) | 2111 (1339–3165) |
| Taita taveta | 6148 (3205–9900) | 6627 (4297–9601) | 1340 (734–2145) | 1524 (980–2213) | 2068 (561–3866) | 5724 (1771–11084) | 2695 (1835–3781) |
| Tana River | 7429 (3991–12028) | 7930 (5032–11719) | 1608 (892–2585) | 1830 (1154–2692) | 2640 (933–4830) | 6906 (2448–13289) | 3243 (2155–4649) |
| Tharaka-nithi | 5701 (3163–9059) | 6064 (3967–8776) | 1231 (704–1947) | 1401 (908–2036) | 2063 (818–3708) | 5296 (2019–9900) | 2485 (1699–3470) |
| Trans Nzoia | 3569 (1841–5995) | 3840 (2382–5855) | 777 (412–1292) | 884 (541–1341) | 1212 (354–2359) | 3322 (1074–6592) | 1563 (1015–2301) |
| Turkana | 5684 (3041–9498) | 6026 (3745–9176) | 1225 (662–2023) | 1393 (856–2114) | 2095 (766–3881) | 5277 (1950–10191) | 2474 (1592–3674) |
| Uasin Gishu | 4792 (2741–7579) | 5036 (3315–7300) | 1026 (597–1622) | 1168 (760–1695) | 1845 (832–3196) | 4443 (1794–8166) | 2078 (1413–2918) |
| Vihiga | 7625 (4101–12582) | 8077 (4942–12475) | 1642 (894–2709) | 1868 (1118–2877) | 2822 (1134–5198) | 7079 (2653–13834) | 3317 (2101–4947) |
| Wajir | 6980 (3811–11963) | 7233 (4372–11618) | 1480 (813–2535) | 1686 (998–2722) | 2876 (1294–5379) | 6457 (2603–12776) | 3009 (1860–4775) |
| West Pokot | 3250 (1626–5490) | 3493 (2064–5478) | 707 (362–1195) | 804 (471–1255) | 1110 (300–2177) | 3024 (958–5978) | 1423 (876–2151) |
| UI, uncertainty interval. No admission/recommendation denotes HUS-defined SARI episodes with neither actual admission nor recommendation for hospitalization. | | | | | | | |

**Supplemental Table 6. Influenza positivity inputs used for attribution of HUS-defined SARI, Kenya, 2017–2019**

| **Input type** | **Sentinel site / model input** | **Samples tested** | **Samples positive** | **Influenza positivity (%)** |
| --- | --- | --- | --- | --- |
| Observed surveillance site | Kakamega | 152 | 18 | 11.8 |
| Observed surveillance site | Kenyatta National Hospital (Nairobi) | 293 | 28 | 9.6 |
| Observed surveillance site | Marsabit | 76 | 5 | 6.6 |
| Observed surveillance site | Mombasa | 217 | 22 | 10.1 |
| Observed surveillance site | Nakuru | 283 | 39 | 13.8 |
| Observed surveillance site | Nyeri | 256 | 37 | 14.5 |
| Observed surveillance site | Siaya | 244 | 23 | 9.4 |
| Derived model input | Kenya national surveillance total | 1521 | 172 | 11.3 |
| Derived model input | Pooled Kakamega-Nakuru-Siaya surveillance input | 679 | 80 | 11.8 |
| Derived model input | Eastern proxy composite | 262.25 | 31.5 | 12.0 |
| Observed surveillance total: 1,521 specimens tested and 172 influenza-positive (11.3%). Derived model inputs are analytic composites rather than additional observed surveillance sites; effective tested/positive values can therefore be non-integers. All-age positivity from the assigned source was applied across age groups in the primary burden model. | | | | |

**Supplemental Table 7A. Influenza-associated HUS-defined SARI episode rates per 100,000 population at national and county level by age group, Kenya, 2018**

| **National/County** | **<2 years** | **2–4 years** | **5–14 years** | **15–49 years** | **50–64 years** | **≥65 years** | **All age groups** |
| --- | --- | --- | --- | --- | --- | --- | --- |
| KENYA | 853 (549–1254) | 868 (622–1204) | 179 (115–267) | 204 (143–282) | 381 (193–635) | 787 (346–1424) | 365 (270–493) |
| Baringo | 831 (461–1348) | 846 (511–1314) | 174 (97–285) | 198 (117–309) | 372 (168–655) | 767 (303–1492) | 356 (220–542) |
| Bomet | 771 (424–1255) | 785 (466–1225) | 161 (89–266) | 184 (107–289) | 345 (156–622) | 711 (284–1383) | 330 (200–505) |
| Bungoma | 825 (427–1428) | 839 (463–1395) | 173 (89–299) | 197 (107–327) | 369 (157–697) | 760 (291–1521) | 353 (197–576) |
| Busia | 1239 (641–2144) | 1260 (704–2071) | 259 (137–445) | 296 (162–486) | 554 (237–1044) | 1143 (440–2293) | 530 (300–856) |
| Elgeyo-Marakwet | 843 (484–1350) | 857 (532–1304) | 176 (101–284) | 201 (122–309) | 377 (174–673) | 777 (313–1489) | 361 (229–539) |
| Embu | 987 (549–1648) | 1004 (602–1613) | 207 (115–344) | 235 (137–380) | 441 (202–823) | 910 (361–1792) | 422 (256–666) |
| Garissa | 575 (192–1282) | 584 (201–1261) | 120 (39–267) | 137 (46–298) | 257 (77–620) | 530 (146–1335) | 246 (85–523) |
| Homabay | 1192 (669–2070) | 1212 (723–2000) | 249 (139–427) | 284 (169–471) | 533 (242–996) | 1099 (446–2203) | 510 (314–828) |
| Isiolo | 631 (214–1356) | 641 (220–1338) | 132 (43–283) | 150 (51–314) | 282 (83–649) | 582 (159–1416) | 270 (94–553) |
| Kajiado | 905 (513–1433) | 920 (573–1390) | 189 (110–304) | 216 (133–329) | 404 (187–708) | 834 (342–1596) | 387 (247–575) |
| Kakamega | 871 (400–1606) | 867 (448–1475) | 382 (186–679) | 312 (159–543) | 517 (123–1149) | 1339 (354–3106) | 488 (276–782) |
| Kericho | 814 (449–1334) | 828 (495–1279) | 170 (95–284) | 194 (114–303) | 364 (164–648) | 751 (299–1455) | 348 (213–529) |
| Kiambu | 952 (569–1549) | 968 (631–1501) | 199 (119–326) | 227 (146–348) | 425 (205–762) | 878 (368–1694) | 407 (272–614) |
| Kilifi | 771 (436–1304) | 784 (481–1265) | 161 (91–275) | 184 (110–296) | 345 (157–640) | 711 (288–1420) | 330 (205–522) |
| Kirinyaga | 1085 (641–1782) | 1104 (701–1726) | 227 (135–376) | 259 (162–408) | 485 (234–885) | 1001 (422–1950) | 464 (305–715) |
| Kisii | 577 (310–1010) | 587 (336–997) | 121 (64–212) | 138 (78–231) | 258 (114–498) | 532 (210–1096) | 247 (144–409) |
| Kisumu | 904 (479–1604) | 919 (516–1579) | 189 (100–337) | 216 (119–367) | 404 (177–786) | 834 (323–1714) | 387 (220–650) |
| Kitui | 655 (355–1100) | 666 (387–1081) | 137 (74–231) | 156 (88–256) | 293 (131–544) | 604 (232–1197) | 280 (165–442) |
| Kwale | 753 (422–1281) | 765 (466–1247) | 158 (89–273) | 180 (107–293) | 336 (154–629) | 694 (276–1400) | 322 (199–518) |
| Laikipia | 677 (377–1115) | 689 (415–1080) | 142 (79–238) | 162 (96–256) | 303 (138–546) | 625 (251–1229) | 290 (179–446) |
| Lamu | 1069 (611–1781) | 1087 (672–1748) | 224 (126–382) | 255 (154–414) | 478 (222–891) | 986 (404–1970) | 457 (288–722) |
| Machakos | 755 (430–1241) | 768 (471–1208) | 158 (89–262) | 180 (108–284) | 337 (155–607) | 696 (282–1348) | 323 (201–497) |
| Makueni | 765 (418–1287) | 778 (456–1262) | 160 (87–273) | 183 (104–297) | 342 (156–632) | 706 (277–1401) | 327 (194–523) |
| Mandera | 650 (209–1468) | 661 (219–1469) | 136 (43–309) | 155 (50–344) | 290 (83–708) | 599 (159–1525) | 278 (93–604) |
| Marsabit | 496 (163–1071) | 505 (171–1060) | 104 (34–225) | 118 (40–252) | 222 (65–523) | 458 (123–1127) | 212 (73–441) |
| Meru | 1190 (672–1934) | 1210 (743–1873) | 249 (141–402) | 284 (169–446) | 532 (246–968) | 1097 (440–2112) | 509 (318–770) |
| Migori | 1119 (628–1922) | 1138 (681–1866) | 234 (130–402) | 267 (156–439) | 500 (228–936) | 1032 (425–2079) | 479 (293–771) |
| Mombasa | 858 (484–1455) | 872 (532–1410) | 179 (101–308) | 205 (122–330) | 383 (176–715) | 791 (322–1582) | 367 (229–582) |
| Muranga | 1059 (636–1715) | 1077 (707–1648) | 222 (134–361) | 253 (163–391) | 473 (228–849) | 976 (413–1888) | 453 (308–684) |
| Nairobi | 577 (332–943) | 586 (369–905) | 121 (70–198) | 138 (85–216) | 258 (122–468) | 532 (220–1029) | 247 (159–376) |
| Nakuru | 2210 (1226–3606) | 2290 (1373–3576) | 280 (97–537) | 336 (175–524) | 702 (173–1384) | 871 (0–2477) | 796 (496–1178) |
| Nandi | 762 (433–1227) | 775 (478–1188) | 160 (90–260) | 182 (110–283) | 341 (155–607) | 703 (288–1351) | 326 (206–493) |
| Narok | 891 (512–1432) | 906 (565–1386) | 187 (108–303) | 213 (130–325) | 398 (185–699) | 822 (331–1562) | 381 (242–573) |
| Nyamira | 672 (368–1166) | 683 (405–1132) | 141 (77–243) | 160 (93–264) | 300 (134–564) | 619 (248–1250) | 287 (173–466) |
| Nyandarua | 1463 (880–2373) | 1488 (969–2285) | 306 (184–499) | 349 (227–536) | 654 (315–1173) | 1350 (569–2608) | 626 (422–940) |
| Nyeri | 1326 (808–2120) | 1349 (898–2044) | 278 (170–445) | 316 (208–484) | 593 (290–1054) | 1223 (519–2314) | 567 (390–846) |
| Samburu | 935 (514–1551) | 951 (563–1499) | 196 (108–323) | 223 (130–351) | 418 (189–759) | 862 (343–1698) | 400 (242–620) |
| Siaya | 557 (183–1330) | 702 (378–1239) | 100 (42–196) | 185 (98–324) | 363 (78–870) | 766 (91–1950) | 283 (162–475) |
| Taita taveta | 965 (561–1614) | 982 (611–1559) | 202 (116–343) | 230 (141–367) | 431 (203–799) | 890 (364–1766) | 413 (263–645) |
| Tana River | 1094 (606–1869) | 1112 (675–1807) | 229 (129–395) | 261 (155–430) | 489 (225–921) | 1009 (407–2012) | 468 (288–755) |
| Tharaka-nithi | 984 (558–1627) | 1001 (607–1563) | 206 (115–337) | 235 (139–372) | 440 (201–805) | 908 (362–1760) | 421 (261–649) |
| Trans Nzoia | 776 (435–1265) | 789 (473–1221) | 162 (90–267) | 185 (111–287) | 347 (157–615) | 716 (285–1395) | 332 (204–504) |
| Turkana | 559 (187–1226) | 568 (193–1197) | 117 (38–257) | 133 (44–281) | 250 (74–586) | 515 (142–1286) | 239 (82–496) |
| Uasin Gishu | 885 (502–1414) | 900 (555–1362) | 185 (105–297) | 211 (128–325) | 395 (182–699) | 816 (327–1576) | 378 (238–562) |
| Vihiga | 1278 (638–2273) | 1300 (693–2227) | 267 (135–480) | 305 (159–524) | 571 (239–1111) | 1178 (442–2464) | 546 (296–925) |
| Wajir | 577 (188–1344) | 586 (198–1321) | 121 (39–279) | 138 (46–310) | 258 (75–639) | 532 (142–1386) | 247 (84–549) |
| West Pokot | 702 (381–1172) | 714 (415–1133) | 147 (81–247) | 168 (97–267) | 314 (140–568) | 648 (254–1276) | 300 (179–469) |

**Supplemental Table 7B. Influenza-associated SARI episode rates with actual admission or recommendation for hospitalization per 100,000 population at national and county level by age group, Kenya, 2018**

| **National/County** | **<2 years** | **2–4 years** | **5–14 years** | **15–49 years** | **50–64 years** | **≥65 years** | **All age groups** |
| --- | --- | --- | --- | --- | --- | --- | --- |
| KENYA | 255 (135–423) | 232 (143–356) | 50 (25–84) | 57 (32–90) | 164 (45–340) | 232 (25–630) | 104 (72–149) |
| Baringo | 253 (116–462) | 230 (120–397) | 49 (21–92) | 56 (27–100) | 162 (41–362) | 229 (24–658) | 103 (57–171) |
| Bomet | 224 (94–424) | 204 (96–366) | 43 (18–85) | 50 (22–92) | 143 (35–331) | 203 (21–571) | 91 (46–156) |
| Bungoma | 185 (76–365) | 168 (77–313) | 36 (14–72) | 41 (18–79) | 118 (28–281) | 167 (17–501) | 76 (37–134) |
| Busia | 472 (206–903) | 430 (215–771) | 92 (39–178) | 105 (49–195) | 303 (75–701) | 428 (45–1246) | 193 (102–330) |
| Elgeyo-Marakwet | 359 (171–647) | 327 (177–549) | 70 (32–128) | 80 (41–137) | 230 (60–499) | 326 (33–894) | 147 (87–234) |
| Embu | 388 (178–734) | 354 (184–633) | 75 (33–147) | 86 (41–159) | 249 (63–576) | 352 (37–1027) | 159 (88–273) |
| Garissa | 90 (0–273) | 82 (0–242) | 17 (0–55) | 20 (0–61) | 58 (0–203) | 81 (0–333) | 37 (0–107) |
| Homabay | 368 (173–705) | 335 (176–602) | 71 (32–138) | 82 (40–149) | 236 (59–550) | 334 (35–974) | 151 (86–255) |
| Isiolo | 182 (54–441) | 166 (52–374) | 35 (10–86) | 40 (12–93) | 117 (22–326) | 165 (16–561) | 74 (24–165) |
| Kajiado | 256 (116–463) | 233 (122–394) | 50 (22–92) | 57 (28–100) | 164 (42–360) | 232 (24–651) | 105 (59–169) |
| Kakamega | 254 (68–553) | 313 (127–592) | 95 (37–187) | 47 (12–104) | 189 (0–574) | 460 (0–1599) | 131 (67–228) |
| Kericho | 243 (108–455) | 221 (112–390) | 47 (21–91) | 54 (26–97) | 156 (40–353) | 220 (23–633) | 99 (54–169) |
| Kiambu | 327 (157–607) | 298 (164–514) | 64 (29–121) | 73 (37–131) | 210 (56–478) | 297 (32–851) | 134 (79–221) |
| Kilifi | 199 (90–388) | 182 (91–331) | 39 (17–78) | 44 (21–83) | 128 (32–300) | 181 (19–543) | 82 (44–142) |
| Kirinyaga | 168 (0–536) | 153 (0–477) | 33 (0–106) | 37 (0–116) | 107 (0–378) | 152 (0–632) | 69 (0–208) |
| Kisii | 168 (72–339) | 153 (71–295) | 33 (13–67) | 37 (16–74) | 108 (26–264) | 153 (16–471) | 69 (35–127) |
| Kisumu | 316 (138–633) | 288 (138–554) | 61 (26–127) | 70 (32–138) | 202 (49–491) | 286 (29–870) | 129 (67–237) |
| Kitui | 171 (74–330) | 156 (75–289) | 33 (14–67) | 38 (17–73) | 110 (27–255) | 155 (16–457) | 70 (36–124) |
| Kwale | 207 (91–409) | 188 (93–359) | 40 (17–82) | 46 (21–88) | 132 (33–316) | 187 (21–560) | 84 (44–153) |
| Laikipia | 225 (101–423) | 205 (103–365) | 44 (19–85) | 50 (24–91) | 144 (37–325) | 204 (21–578) | 92 (49–157) |
| Lamu | 333 (155–636) | 303 (155–550) | 65 (28–128) | 74 (36–139) | 214 (55–505) | 302 (32–904) | 136 (75–237) |
| Machakos | 187 (81–361) | 170 (82–308) | 36 (15–72) | 42 (19–78) | 120 (29–278) | 169 (18–495) | 76 (40–133) |
| Makueni | 224 (97–428) | 204 (100–372) | 44 (18–86) | 50 (23–93) | 144 (36–338) | 203 (21–593) | 92 (48–159) |
| Mandera | 172 (0–472) | 157 (0–415) | 33 (0–93) | 38 (0–101) | 110 (0–351) | 156 (0–585) | 70 (0–181) |
| Marsabit | 146 (38–350) | 133 (37–311) | 28 (7–69) | 32 (9–77) | 93 (16–263) | 132 (11–451) | 60 (17–133) |
| Meru | 500 (233–915) | 455 (242–771) | 97 (44–180) | 111 (55–195) | 321 (81–715) | 454 (49–1285) | 205 (118–332) |
| Migori | 380 (174–735) | 346 (178–622) | 74 (32–143) | 84 (41–155) | 244 (60–566) | 345 (36–1015) | 155 (86–266) |
| Mombasa | 298 (132–574) | 271 (136–490) | 58 (24–114) | 66 (31–124) | 191 (49–441) | 270 (28–795) | 122 (65–213) |
| Muranga | 375 (182–688) | 341 (186–587) | 73 (33–137) | 83 (43–146) | 240 (62–541) | 340 (37–965) | 153 (91–251) |
| Nairobi | 171 (77–321) | 156 (80–274) | 33 (15–64) | 38 (18–70) | 109 (28–251) | 155 (16–457) | 70 (39–118) |
| Nakuru | 790 (286–1570) | 535 (225–967) | 84 (0–214) | 149 (56–253) | 169 (0–650) | 196 (0–1020) | 248 (127–397) |
| Nandi | 228 (104–422) | 207 (109–361) | 44 (20–83) | 51 (25–92) | 146 (36–329) | 206 (21–596) | 93 (52–155) |
| Narok | 277 (125–508) | 252 (134–430) | 54 (24–101) | 62 (30–109) | 178 (46–392) | 251 (27–710) | 113 (64–185) |
| Nyamira | 195 (76–445) | 178 (75–386) | 38 (14–87) | 43 (17–96) | 125 (27–334) | 177 (18–576) | 80 (35–170) |
| Nyandarua | 348 (122–755) | 317 (123–651) | 68 (23–150) | 77 (29–163) | 223 (49–560) | 316 (31–1001) | 142 (58–279) |
| Nyeri | 440 (203–807) | 400 (214–692) | 85 (39–161) | 98 (49–173) | 282 (73–641) | 399 (43–1144) | 180 (103–294) |
| Samburu | 235 (90–470) | 214 (92–414) | 46 (17–94) | 52 (21–102) | 150 (33–366) | 213 (21–642) | 96 (44–177) |
| Siaya | 144 (23–347) | 199 (73–414) | 32 (3–89) | 65 (22–144) | 288 (24–773) | 160 (0–564) | 93 (45–173) |
| Taita taveta | 351 (167–664) | 319 (171–566) | 68 (30–132) | 78 (39–144) | 225 (59–513) | 318 (34–935) | 143 (82–243) |
| Tana River | 351 (162–678) | 319 (166–580) | 68 (30–138) | 78 (38–147) | 225 (58–520) | 318 (34–941) | 143 (80–252) |
| Tharaka-nithi | 300 (136–560) | 273 (140–478) | 58 (25–111) | 67 (31–121) | 192 (48–442) | 272 (28–775) | 123 (67–207) |
| Trans Nzoia | 276 (124–504) | 252 (128–428) | 54 (23–100) | 61 (29–108) | 177 (44–387) | 251 (26–711) | 113 (62–183) |
| Turkana | 161 (44–404) | 147 (44–347) | 31 (9–79) | 36 (10–87) | 103 (19–304) | 146 (13–517) | 66 (20–152) |
| Uasin Gishu | 214 (91–395) | 195 (95–340) | 42 (17–78) | 48 (22–86) | 137 (33–307) | 194 (19–551) | 88 (45–146) |
| Vihiga | 363 (153–739) | 330 (153–629) | 70 (29–146) | 81 (36–159) | 232 (58–566) | 329 (33–1012) | 148 (73–275) |
| Wajir | 88 (21–257) | 80 (20–226) | 17 (4–49) | 20 (5–55) | 56 (9–186) | 80 (6–321) | 36 (9–99) |
| West Pokot | 247 (110–468) | 225 (115–399) | 48 (21–92) | 55 (26–100) | 158 (39–361) | 224 (24–642) | 101 (55–172) |

**Supplemental Table 7C. Influenza-associated SARI without admission/recommendation episode rates per 100,000 population at national and county level by age group, Kenya, 2018**

| **National/County** | **<2 years** | **2–4 years** | **5–14 years** | **15–49 years** | **50–64 years** | **≥65 years** | **All age groups** |
| --- | --- | --- | --- | --- | --- | --- | --- |
| KENYA | 598 (350–937) | 635 (436–913) | 129 (77–201) | 147 (99–207) | 218 (95–373) | 555 (216–1035) | 261 (187–359) |
| Baringo | 578 (291–995) | 615 (358–982) | 125 (64–212) | 142 (81–226) | 209 (79–394) | 537 (191–1070) | 252 (151–390) |
| Bomet | 548 (276–949) | 581 (329–938) | 118 (60–202) | 134 (75–217) | 201 (75–384) | 509 (178–1021) | 238 (139–374) |
| Bungoma | 640 (316–1142) | 670 (363–1135) | 137 (68–243) | 156 (84–259) | 250 (104–481) | 593 (222–1171) | 277 (153–455) |
| Busia | 767 (342–1433) | 830 (439–1412) | 168 (78–308) | 191 (99–325) | 251 (53–534) | 714 (197–1534) | 337 (184–557) |
| Elgeyo-Marakwet | 484 (215–866) | 531 (303–854) | 107 (51–187) | 121 (68–195) | 147 (4–325) | 452 (95–963) | 214 (128–331) |
| Embu | 599 (268–1090) | 650 (362–1087) | 131 (62–234) | 149 (80–246) | 192 (26–413) | 558 (144–1195) | 263 (151–425) |
| Garissa | 485 (160–1116) | 503 (171–1108) | 103 (33–236) | 117 (39–260) | 199 (56–501) | 449 (120–1157) | 209 (71–459) |
| Homabay | 824 (416–1488) | 877 (506–1484) | 178 (93–317) | 202 (117–338) | 297 (113–591) | 765 (275–1581) | 359 (216–589) |
| Isiolo | 449 (146–997) | 476 (160–1010) | 97 (30–214) | 110 (37–234) | 165 (44–398) | 417 (106–1043) | 195 (67–406) |
| Kajiado | 648 (343–1091) | 687 (413–1070) | 140 (75–234) | 159 (95–249) | 240 (98–445) | 602 (224–1176) | 282 (176–428) |
| Kakamega | 617 (251–1213) | 554 (256–1029) | 287 (122–544) | 265 (132–467) | 328 (66–749) | 879 (230–1925) | 357 (196–588) |
| Kericho | 571 (285–982) | 607 (348–962) | 123 (63–212) | 140 (80–223) | 208 (78–390) | 530 (188–1050) | 249 (147–387) |
| Kiambu | 625 (321–1095) | 670 (409–1078) | 136 (72–234) | 154 (93–243) | 216 (64–426) | 581 (191–1179) | 273 (173–427) |
| Kilifi | 572 (303–1003) | 603 (361–986) | 123 (66–216) | 140 (81–228) | 217 (90–414) | 530 (204–1073) | 248 (152–397) |
| Kirinyaga | 918 (462–1569) | 951 (541–1531) | 195 (101–331) | 222 (125–358) | 378 (132–716) | 849 (314–1690) | 396 (230–626) |
| Kisii | 409 (197–750) | 433 (239–758) | 88 (44–160) | 100 (54–173) | 150 (54–303) | 379 (131–794) | 178 (101–301) |
| Kisumu | 588 (263–1118) | 632 (333–1111) | 128 (60–241) | 145 (77–255) | 202 (52–430) | 547 (166–1182) | 257 (141–444) |
| Kitui | 484 (242–845) | 511 (288–836) | 104 (53–181) | 118 (65–194) | 183 (73–350) | 449 (165–893) | 210 (122–336) |
| Kwale | 546 (279–974) | 577 (339–959) | 117 (62–210) | 134 (77–220) | 204 (79–395) | 507 (182–1047) | 237 (143–387) |
| Laikipia | 452 (219–795) | 484 (280–787) | 98 (50–171) | 112 (63–183) | 159 (48–315) | 421 (136–869) | 198 (117–314) |
| Lamu | 735 (378–1309) | 784 (464–1299) | 159 (83–283) | 181 (105–300) | 264 (94–531) | 683 (239–1416) | 321 (196–517) |
| Machakos | 568 (298–970) | 598 (355–959) | 122 (65–207) | 139 (80–222) | 218 (90–406) | 527 (199–1028) | 246 (150–385) |
| Makueni | 541 (269–956) | 574 (324–949) | 117 (59–206) | 133 (73–221) | 198 (73–389) | 502 (177–1029) | 236 (135–381) |
| Mandera | 478 (143–1138) | 504 (161–1164) | 103 (31–242) | 117 (37–268) | 180 (41–464) | 443 (105–1173) | 207 (67–467) |
| Marsabit | 351 (112–805) | 372 (124–813) | 76 (24–171) | 86 (28–189) | 128 (33–323) | 326 (82–828) | 153 (51–328) |
| Meru | 690 (305–1250) | 755 (426–1221) | 152 (73–269) | 173 (96–282) | 211 (18–466) | 644 (145–1382) | 304 (179–475) |
| Migori | 739 (358–1351) | 792 (449–1349) | 160 (81–288) | 182 (102–307) | 257 (81–529) | 687 (229–1450) | 323 (189–533) |
| Mombasa | 560 (270–1018) | 601 (348–1015) | 122 (61–221) | 138 (78–230) | 193 (51–403) | 521 (164–1108) | 245 (146–403) |
| Muranga | 684 (352–1202) | 735 (452–1179) | 149 (79–258) | 169 (103–270) | 233 (67–469) | 636 (210–1290) | 299 (193–467) |
| Nairobi | 406 (212–703) | 431 (259–683) | 88 (47–150) | 100 (59–159) | 148 (57–284) | 377 (135–746) | 177 (110–275) |
| Nakuru | 1420 (617–2605) | 1755 (990–2889) | 196 (61–382) | 187 (81–321) | 533 (107–1122) | 675 (0–2127) | 548 (324–854) |
| Nandi | 535 (274–911) | 568 (336–901) | 115 (60–197) | 131 (77–208) | 195 (72–369) | 497 (177–976) | 233 (142–360) |
| Narok | 614 (313–1043) | 654 (391–1027) | 133 (70–225) | 151 (88–235) | 221 (80–413) | 570 (199–1128) | 268 (164–408) |
| Nyamira | 476 (212–873) | 505 (267–864) | 103 (49–187) | 117 (62–198) | 175 (40–351) | 442 (137–921) | 207 (113–347) |
| Nyandarua | 1115 (588–1903) | 1171 (716–1859) | 239 (131–402) | 272 (166–430) | 431 (167–821) | 1034 (396–2017) | 483 (305–751) |
| Nyeri | 887 (475–1541) | 949 (594–1497) | 192 (106–329) | 219 (136–347) | 311 (98–613) | 825 (286–1653) | 387 (254–595) |
| Samburu | 700 (360–1212) | 737 (421–1193) | 150 (77–257) | 171 (95–277) | 268 (106–508) | 650 (237–1291) | 304 (178–481) |
| Siaya | 412 (85–1095) | 503 (248–940) | 68 (21–147) | 120 (57–219) | 74 (0–292) | 606 (37–1694) | 190 (105–328) |
| Taita taveta | 615 (297–1108) | 663 (388–1094) | 134 (67–240) | 152 (88–249) | 207 (56–427) | 572 (171–1209) | 269 (165–431) |
| Tana River | 743 (366–1359) | 793 (455–1325) | 161 (82–289) | 183 (104–305) | 264 (90–534) | 691 (233–1442) | 324 (193–534) |
| Tharaka-nithi | 684 (344–1185) | 728 (424–1169) | 148 (76–255) | 168 (97–273) | 248 (92–480) | 635 (226–1272) | 298 (179–467) |
| Trans Nzoia | 500 (240–882) | 538 (307–863) | 109 (55–191) | 124 (71–200) | 170 (49–342) | 465 (147–970) | 219 (130–343) |
| Turkana | 398 (127–908) | 422 (142–911) | 86 (27–195) | 98 (32–210) | 147 (37–362) | 369 (92–944) | 173 (59–366) |
| Uasin Gishu | 671 (360–1111) | 705 (422–1088) | 144 (78–239) | 164 (98–255) | 258 (111–468) | 622 (239–1209) | 291 (178–439) |
| Vihiga | 915 (425–1688) | 969 (501–1695) | 197 (93–366) | 224 (114–390) | 339 (122–686) | 849 (288–1837) | 398 (211–676) |
| Wajir | 489 (156–1151) | 506 (167–1142) | 104 (33–242) | 118 (39–267) | 201 (57–507) | 452 (118–1172) | 211 (70–472) |
| West Pokot | 455 (215–806) | 489 (269–808) | 99 (48–176) | 113 (61–185) | 155 (41–316) | 423 (128–868) | 199 (114–317) |
| UI, uncertainty interval. Age-specific influenza-associated estimates use all-age influenza positivity from the assigned surveillance site or regional composite. No admission/recommendation denotes the component with neither actual admission nor recommendation for hospitalization. | | | | | | | |

# Supplementary Methods

# Steps and equations used to calculate HUS-defined SARI and influenza-associated SARI, stratified as episodes with actual admission or recommendation for hospitalization and SARI without admission or recommendation for hospitalization.

**Step 1. Calculation of base HUS-defined SARI episode rates**

For the burden model, pooled age-specific HUS-defined SARI episode rates were calculated from participants in the three counties included in the burden analysis as the number of SARI episodes during the 12-month recall period divided by the enrolled analytic population (Eq. 1). Recurrent episodes during the 12-month recall period were retained in estimating the annual HUS-defined SARI episode rate. Rates are shown per 100,000 in the manuscript tables and figures; the equations below use the underlying per-person rates.

*Equation 1: Base HUS-defined SARI episode rate*

$I_{B,a}=\frac{{SARI}_{B,a}}{N_{B,a}}$

Eq. 1

Where:

$I_{B,a}$ = Base HUS-defined SARI episode rate for age group a in the pooled HUS base

${SARI}_{B,a}$ = Number of HUS-defined SARI episodes among enrolled participants in age group a during the 12-month recall period

$N_{B,a}$ = Number of enrolled HUS participants in age group a in the pooled HUS base

Here, B denotes the pooled HUS base (Kakamega, Nakuru, and Siaya), and a denotes age group.

**Step 2a: Calculation of a relative geographic adjustment factor for SARI burden by county**

County geographic adjustment factors were derived from lower respiratory infection (LRI) disability-adjusted life years (DALYs) from GBD 2016 [1,2]. The GBD Kenya analysis synthesized Kenyan mortality, morbidity, surveillance, survey, census, registry, and published data, used localized covariates, and produced modeled estimates for all 47 counties. The resulting county-specific modeled LRI DALY rates served as the geographic scaling input for county-level extrapolation.

For each county, uncertainty in years of life lost due to premature death (YLL) and years lived with disability (YLD) was propagated with 10,000 joint draws. YLL and YLD were combined and divided by the corresponding GBD population denominator to obtain an LRI DALY rate. Within each iteration, the pooled base DALY rate for Kakamega, Nakuru, and Siaya was calculated coherently using one shared denominator, and each county adjustment factor was calculated relative to that shared base.

The central base DALY rate was calculated from Kakamega, Nakuru, and Siaya combined as the sum of YLL and YLD across the three counties divided by their combined GBD population denominator. Each county's central adjustment factor was its LRI DALY rate divided by the combined Kakamega-Nakuru-Siaya LRI DALY rate (Eq. 3). This assumes that relative geographic variation in HUS-defined SARI is reasonably approximated by relative geographic variation in modeled LRI DALY burden.

*Equations 2 & 3: Calculation of LRI DALY rates and adjustment factor by county*

$$D_{y}=\frac{({YLL}_{y}+{YLD}_{y})}{P_{GBD,y}}$$

Eq. 2

$${Adj}_{y}=\frac{D_{y}}{D_{B}}$$

Eq. 3

Where:

$D_{y}$ = LRI DALY rate in county y

${YLL}_{y}$ = Years of life lost due to premature death in county y

${YLD}_{y}$ = Years lived with disability in county y

$P_{GBD,y}$ = GBD population denominator for county y

${Adj}_{y}$ = Relative LRI DALY adjustment factor for county y

$D_{B}$ = Pooled LRI DALY rate for the HUS base counties (Kakamega, Nakuru, and Siaya)

**Step 2b: Calculation of the HUS-defined SARI episode rate by county**

The HUS-defined SARI episode rate in each county was calculated by multiplying the base age-specific HUS rate for each age group by the relative LRI DALY adjustment factor for that county.

*Equation 4: HUS-defined SARI episode rate by county*

$$I_{y,a}=I_{B,a}\times{Adj}_{y}$$

Eq. 4

Where:

$I_{y,a}$ = HUS-defined SARI episode rate in county y for age group a

**Step 3: Calculation of SARI with actual admission or recommendation for hospitalization**

The HUS supplied age-specific proportions of HUS-defined SARI episodes that resulted in either actual hospital admission or a healthcare-professional recommendation for hospitalization. For geographic extrapolation, KDHS 2014 under-five ARI care-seeking data [3] were used to construct a relative county modifier. For each county, the weighted care-seeking probability was divided by the weighted pooled probability in Kakamega, Nakuru, and Siaya.

Because comparable county-specific estimates were unavailable for older age groups, this relative geographic pattern was applied across age strata, while age-specific admission-or-recommendation proportions remained HUS-derived. This assumes that relative geographic variation in under-five ARI care seeking is a reasonable proxy for relative geographic variation across ages. Healthcare-seeking behavior varies by age and context [4–6], so the relative geographic pattern may not transfer uniformly across age strata.

The SARI admission-or-recommendation rate in each county was calculated by multiplying the county total HUS-defined SARI rate by the HUS age-specific admission-or-recommendation proportion and the relative KDHS county healthcare-seeking modifier.

*Equation 5: SARI with actual admission or recommendation for hospitalization by county*

$$I_{AR,y,a}=I_{y,a}\times p_{a}\times\frac{{DHS}_{y}}{{DHS}_{B}}$$

Eq. 5

Where:

$I_{AR,y,a}$ = Rate of HUS-defined SARI with actual admission or recommendation for hospitalization in county y and age group a

$p_{a}$ = Age-specific proportion of HUS-defined SARI episodes with actual admission or recommendation for hospitalization in age group a in the pooled HUS base

${DHS}_{y}$ = Weighted proportion of under-five ARI episodes seeking care in county y (KDHS)

${DHS}_{B}$ = Weighted pooled proportion of under-five ARI episodes seeking care in Kakamega, Nakuru, and Siaya combined (KDHS)

***Step 4: Calculation of SARI without admission/recommendation***

The SARI without admission/recommendation rate was calculated as the difference between the total HUS-defined SARI rate in the county and the rate with actual admission or recommendation for hospitalization.

*Equation 6: SARI without admission/recommendation rate by county*

$$I_{NAR,y,a}=I_{y,a}-I_{AR,y,a}$$

Eq. 6

Where:

$I_{NAR,y,a}$ = HUS-defined SARI rate without admission/recommendation in county y and age group a

**Step 5: Calculation of influenza-associated SARI**

Influenza attribution was derived from seven observed sentinel surveillance sites: Siaya, Kakamega, Mombasa, Nakuru, Nyeri, Kenyatta National Hospital, and Marsabit. At sentinel sites, surveillance SARI was defined as acute respiratory infection with fever or history of fever and cough with recent onset and requiring hospitalization [7]. This surveillance definition is narrower than the HUS community construct.

Each county was assigned an observed surveillance site or predefined regional composite. The average annual all-age positivity from 2017–2019 for the assigned source was applied to both the admission-or-recommendation and no-admission/recommendation components. The Eastern composite was derived from Mombasa, Nakuru, Nyeri, and Kenyatta. Age-specific influenza positivity was examined, but older-age sampling was sparse: across the seven sites only 11 raw specimens were tested among persons aged 50–64 years and 9 among persons aged ≥65 years, and site-specific denominators remained small even after combining all persons aged ≥5 years. Age-specific site positivity was therefore considered insufficiently stable for county attribution, and the assigned all-age positivity was applied across age groups. This retains geographic variation in influenza circulation but assumes that positivity within an assigned site or region is applicable across ages.

*Equation 7: Influenza-associated SARI with actual admission or recommendation for hospitalization by county*

$$I_{F,AR,y,a}=I_{AR,y,a}\times F_{y}$$

Eq. 7

Where:

$I_{F,AR,y,a}$ = Influenza-associated SARI rate with actual admission or recommendation for hospitalization in county y and age group a

$F_{y}$ = Assigned all-age influenza positivity for county y

*Equation 8: No admission/recommendation influenza-associated SARI by county*

$$I_{F,NAR,y,a}=I_{NAR,y,a}\times F_{y}$$

Eq. 8

Where:

$I_{F,NAR,y,a}$ = Influenza-associated SARI rate without admission/recommendation in county y and age group a

$F_{y}$ = Assigned all-age influenza positivity for county y

**Step 6: Calculation of annual numbers of HUS-defined SARI and influenza-associated SARI episodes**

Annual episode numbers were obtained by multiplying age-specific county rates by projected 2018 age-specific county populations. These populations were reconstructed from the 2009 Kenya Population and Housing Census using the annual growth assumptions used in the original burden analysis [8].

*Equations 9 to 12: Annual numbers of HUS-defined SARI and influenza-associated SARI episodes*

$$N_{AR,y,a}=I_{AR,y,a}\times{Pop}_{y,a}$$

Eq. 9

$$N_{NAR,y,a}=I_{NAR,y,a}\times{Pop}_{y,a}$$

Eq. 10

$$N_{F,AR,y,a}=I_{F,AR,y,a}\times{Pop}_{y,a}$$

Eq. 11

$$N_{F,NAR,y,a}=I_{F,NAR,y,a}\times{Pop}_{y,a}$$

Eq. 12

Where:

$N_{AR,y,a}$ = Number of HUS-defined SARI episodes with actual admission or recommendation for hospitalization in county y and age group a

$N_{NAR,y,a}$ = Number of HUS-defined SARI episodes without admission/recommendation in county y and age group a

$N_{F,AR,y,a}$ = Number of influenza-associated SARI episodes with actual admission or recommendation for hospitalization in county y and age group a

$N_{F,NAR,y,a}$ = Number of influenza-associated SARI episodes without admission/recommendation in county y and age group a

${Pop}_{y,a}$ = Projected 2018 population in county y and age group a

**Uncertainty intervals**

Central point estimates were calculated from observed HUS quantities and central GBD, KDHS, and influenza inputs. Uncertainty was propagated with 10,000 joint iterations. HUS uncertainty was estimated by a two-stage cluster bootstrap within county: sampled sublocation primary sampling units were resampled with replacement and households were then resampled within selected sublocations; age-specific episode rates and admission-or-recommendation proportions were recalculated in each iteration. KDHS uncertainty used a stratified PSU bootstrap respecting the 2014 survey design (v022 strata, v021 PSU, and sample weights), with county care-seeking probabilities and county-to-pooled-HUS relative modifiers derived jointly. GBD geographic factors were generated jointly so that all county factors in an iteration shared the same pooled Kakamega-Nakuru-Siaya DALY denominator. Influenza positivity uncertainty used bounded source-specific draws, with a single draw shared within an iteration by counties assigned to the same surveillance source and applied across ages. These joint inputs were propagated through the burden model. Adjusted admission-or-recommendation probabilities were bounded at 1 in the primary propagation. The reported 95% UIs are the 2.5th and 97.5th percentiles of the resulting distributions.
